# CNS target engagement of high-dose DHA supplementation in older adults at risk for dementia: a randomised, double-blind, placebo-controlled trial

**DOI:** 10.1016/j.ebiom.2026.106316

**Published:** 2026-06-18

**Authors:** Hussein N. Yassine, Sara Ghasem Pour, Marlene Juarez, Isabella C. Arrelanas, Nada Ali, Dante Dikeman, Ashley Sanchez, Jackson Park, Bilal Kerman, Marlon V. Duro, Isaac Asante, Stan Louie, Naoko Kono, Lina M. D'Orazio, Helena Chui, Wendy J. Mack, Michael G. Harrington, Meredith N. Braskie, Lon S. Schneider

**Affiliations:** a Department of Neurology, Keck School of Medicine, University of Southern California, Los Angeles, CA, USA; b Department of Clinical Pharmacy, Alfred E. Mann School of Pharmacy and Pharmaceutical Sciences, University of Southern California, Los Angeles, CA, USA; c Department of Medicine, Keck School of Medicine, University of Southern California, Los Angeles, CA, USA; d Department of Radiology, Keck School of Medicine, University of Southern California, Los Angeles, CA, USA; e Institute for Technology and Medical Systems (ITEMS), Keck School of Medicine, University of Southern California, Los Angeles, CA, USA; f Department of Population and Public Health Sciences, Keck School of Medicine, University of Southern California, Los Angeles, CA, USA; g Mark and Mary Stevens Neuroimaging and Informatics Institute, Keck School of Medicine, University of Southern California, Los Angeles, CA, USA; h Department of Psychiatry and the Behavioral Sciences, Keck School of Medicine, University of Southern California, Los Angeles, CA, USA

**Keywords:** DHA supplementation, APOE ε4, Alzheimer's disease, Cognition, Brain health, Omega-3 fatty acids, Neuroprotection, Dementia prevention

## Abstract

**Background:**

APOE ε4 carriers have increased Alzheimer's disease risk and altered omega-3 metabolism. No large-scale prevention trials have tested high-dose docosahexaenoic acid (DHA) supplementation specifically in non-demented APOE ε4 carriers with low baseline omega-3 intake for early intervention.

**Methods:**

We conducted a phase IIa 24-month, randomised, double-blind, placebo-controlled trial (NCT03613844) at the University of Southern California between September 2018 and May 2024. Participants aged 55–80 years without dementia, low dietary DHA intake (<200 mg/day), and ≥1 dementia risk factor were stratified by cerebrospinal fluid (CSF) collection willingness into lumbar puncture (LP) or no-LP arms. Within each arm, participants were randomised 1:1 to receive 2 g/day DHA or placebo, stratified by APOE ε4 status. Primary outcome was the 6-month CSF DHA-to-arachidonic acid (AA) ratio change. Secondary and exploratory outcomes included 24-month neuroimaging and cognitive measures.

**Findings:**

Of 739 screened individuals, 365 participants were randomised (181 LP arm, 184 no-LP arm). Mean age was 66.4 years (SD 5.7), 210 (58%) were female, 142 (39%) were Hispanic, 173 (47%) were APOE ε4 carriers. DHA supplementation increased CSF DHA/AA ratio at 6 months vs placebo (0.17 [95% CI 0.15–0.18] vs −0.02 [95% CI −0.04 to −0.0004]; difference 0.19 [95% CI 0.16–0.21]; p < 0.0001), independent of APOE ε4 status (interaction p = 0.71). Dropout was 38%, mainly due to COVID-19. No treatment differences were observed in brain volumes or cognitive performance over 24 months. Adverse events were comparable between groups, with no serious adverse events attributed to treatment.

**Interpretation:**

High-dose DHA achieved CNS target engagement in non-demented older adults with low baseline omega-3 intake, independent of APOE ε4. Despite biochemical target engagement, no differences in cognition or brain structure were observed over 24 months. Future research should prioritise brain DHA metabolism over further supplementation trials.

**Funding:**

10.13039/100000049National Institute on Aging (R01AG057684) and the ADDF (GC-201711-2014197).

**Research in context**

**Evidence before this study**

We searched PubMed, Web of Science, and ClinicalTrials.gov for studies of omega-3 supplementation and cognitive outcomes from 2000 to 2026. Numerous observational studies demonstrated modest associations between higher omega-3 intake and reduced risk for cognitive decline, but randomised trials yielded inconsistent results. Only 5 of 24 randomised controlled clinical trials (21%) in people without dementia showed positive cognitive effects with DHA in a recent systematic review. The 4 randomised clinical trials with established Alzheimer's disease all showed null results for DHA. Previous trials had methodological limitations including inadequate sample sizes, heterogeneous populations, short durations, and suboptimal dosing. Few trials specifically targeted non-demented APOE ε4 carriers with low omega-3 intake or assessed brain penetrance through cerebrospinal fluid biomarkers.

**Added value of this study**

This is a large-scale trial (n = 365) specifically designed for non-demented APOE ε4 carriers with low dietary omega-3 intake. High-dose DHA supplementation (2000 mg daily) achieved robust biochemical target engagement, increasing cerebrospinal fluid DHA by 17% and red blood cell omega-3 index from 4.9% to 11.0%, independent of APOE genotype. Despite confirmed brain DHA delivery, participants showed no benefits for hippocampal volume or cognitive performance over 24 months.

**Implications of all the available evidence**

DHA supplementation alone is insufficient to enhance cognition in non-demented older adults with low omega-3 intake. Studying brain DHA metabolism in APOE ε4 carriers remains valuable because APOE ε4 uniquely reshapes DHA transport, incorporation, and catabolism across the lifespan, through which personalised interventions may achieve the clinical benefit that uniform supplementation has not.

## Introduction

Docosahexaenoic acid (DHA) is a structural component of neuronal membranes, maintains synaptic function and modulates neuroinflammation.^1^, ^2^, ^3^ Lower circulating DHA concentrations are associated with cerebral amyloidosis, cognitive decline and late-onset Alzheimer's disease (AD).^4^, ^5^, ^6^ The APOE ε4 allele, the strongest genetic risk factor for AD, drives accelerated DHA catabolism, presenting with lower plasma and cerebrospinal fluid (CSF) DHA levels in carriers vs non-carriers with AD dementia.^7^, ^8^, ^9^ APOE ε4 carriers face earlier disease onset, accelerated hippocampal atrophy, and potentially reduced DHA responsiveness.^10^^,^^11^

Prior DHA supplementation trials in established AD dementia have reported null outcomes^12^ and prevention trials using ≤1 g/day showed no cognitive benefit,^12^ leaving two critical unknowns: whether early intervention in patients with low omega-3 intake before dementia onset is necessary and whether higher doses achieve sufficient brain delivery. Using ^11^C-DHA positron emission tomography, we observed increased brain DHA incorporation in cognitively healthy younger APOE ε4 compared to non-carriers, suggesting increased brain DHA demand decades before clinical symptom onset.^13^ However, no trials have tested whether high-dose DHA supplementation achieves CNS target engagement in non-demented older adults, particularly APOE ε4 carriers, with low dietary omega-3 intake and dementia risk factors.

We conducted a phase 2a, single-centre trial to establish whether high-dose DHA supplementation (2 g/day) in cognitively unimpaired older adults with low baseline omega-3 intake achieves CNS target engagement (primary outcome: CSF DHA/arachidonic acid (AA) ratio at 6 months). We stratified by APOE ε4 status to test whether genotype modifies DHA intervention effects on CSF DHA/AA before the onset of dementia. Secondary outcomes included 24-month changes in hippocampal volume, cortical thickness, and cognitive performance.

## Methods

### Study design and participants

This is a 24-month, randomised, double-blind, single centre phase IIa placebo-controlled trial conducted at the University of Southern California, USA, from September, 2018 to May, 2024. The study protocol^14^ was registered at ClinicalTrials.gov (NCT03613844) and approved by the University of Southern California Institutional Review Board. All participants provided written informed consent (IRB#s HS-18-00291 and HS-18-00984).

Participants were community-dwelling adults aged 55–80 years recruited through advertisements and referrals. Eligibility criteria included: normal cognition defined by Mini-Mental State Examination score ≥25, Logical Memory II Delayed Recall score 7–18, and Functional Activities Questionnaire score <9; limited dietary DHA intake (<200 mg/day assessed by Food Frequency Questionnaire); and at least one cardiovascular or dementia risk factor including obesity (BMI ≥30 kg/m^2^), hypertension, hyperlipidaemia, or physical inactivity (<3 days exercise per week).

Exclusion criteria comprised: diagnosis of dementia or mild cognitive impairment; omega-3 fatty acid supplement use within 3 months; previous use of AD medications; major psychiatric illness; and contraindications to MRI scanning. Additional exclusion criteria for the lumbar puncture arm included anticoagulant medication use, recent spinal injury or infection, and participant refusal of cerebrospinal fluid collection.

### Randomisation and masking

Participants were initially offered enrolment in the lumbar puncture (LP) arm for CSF biomarker collection. Those declining or medically ineligible for lumbar puncture were offered enrolment in the no-LP arm, creating two parallel study populations with identical intervention protocols.

Within each stratum (LP and no-LP arms), participants underwent randomisation using a computer-generated sequence with stratified permuted blocks. Randomisation was stratified by APOE ε4 carrier status (carrier vs non-carrier) and recruitment site, with block sizes of 2 and 4. The randomisation list was uploaded for use in the REDCap randomisation module. A blinded statistician (WJM) generated the randomisation sequence using SAS 9.4 (SAS Institute, Cary NC); an unblinded statistician (NK) performed the randomisations. Participants, investigators, and outcome assessors remained masked to treatment allocation throughout the study.

### Procedures

Participants were randomised 1:1 to receive either high-dose DHA supplementation or matched placebo for 24 months. The active intervention comprised 2000 mg/day DHA delivered as five capsules containing 400 mg DHA each, taken as three capsules with breakfast and two with evening meals, preferably consumed with high-fat foods to optimise absorption. Placebo capsules contained a 1:1 mixture of corn oil and soybean oil with identical appearance, taste, and smell to the active treatment. The 2 g/day DHA dose was selected to exceed thresholds associated with achieving an omega-3 index ≥8–10% and ensure sufficient CSF exposure within 6 months.

All participants received standardised vitamin B-complex supplementation throughout the trial.^15^ Study visits occurred at baseline, 6, 12, 18, and 24 months, with telephone contact at intermediate time points to assess adherence and adverse events. Adherence was monitored by capsule counts at each visit, with adherence ≥80% considered optimal.

### Outcomes

The primary outcome was change in CSF DHA to AA ratio from baseline to 6 months in the LP arm, representing the primary biomarker of brain DHA delivery. Secondary outcomes included changes from baseline to 24 months in: total hippocampal volume measured by deep learning segmentation; cortical thickness in AD-vulnerable regions measured by FreeSurfer analysis; and hippocampal subfield volumes.

Exploratory outcomes comprised: cognitive performance assessed by the Repeatable Battery for the Assessment of Neuropsychological Status (RBANS) total composite score and individual domain scores (immediate memory, delayed memory, visuospatial abilities, language, attention); changes in red blood cell and plasma fatty acid concentrations; and plasma AD biomarkers.

### Laboratory procedures

Fatty acid concentrations were measured in CSF and plasma at baseline and 6 months (LP arm only), and in red blood cells at baseline, 6, and 24 months. CSF was obtained by lumbar puncture performed by trained neurologists using standardised protocols. Fatty acid analysis used gas chromatography-mass spectrometry for CSF and red blood cell samples^15^ and liquid chromatography-mass spectrometry for plasma samples.^16^ The omega-3 index was calculated as (EPA + DHA)/total fatty acids × 100.

### Neuroimaging

MRI scans were acquired at baseline and 24 months using 3.0 T scanners with standardised ADNI-2 protocols to ensure measurement consistency. Structural imaging included high-resolution T1-weighted sequences for morphometric analysis. Cortical thickness and intracranial volume were calculated using FreeSurfer version 5.3. Total hippocampal volume was estimated using HippoDeep deep learning segmentation, whilst hippocampal subfields were segmented using the Automatic Segmentation of Hippocampal Subfields method with population-specific atlases. Quality control procedures included visual inspection by trained raters blinded to treatment assignment and time point.

### Cognitive assessment

Cognitive function was assessed using RBANS at baseline and all follow-up visits in English or Spanish. To minimise practice effects, validated alternate forms were administered at each assessment. Testing was conducted by trained, certified personnel masked to treatment allocation. Raw scores were converted to age-adjusted standard index scores (mean = 100, SD = 15) according to published normative data.^17^

### Statistical analysis

The initial sample size estimation was based on testing the hypothesis that a DHA supplementation effect on CSF DHA would be larger among APOE ε4 non-carriers compared with carriers. The estimation was based on available CSF DHA data rather than the primary outcome of CSF DHA/AA, with the expectation that effect sizes would be comparable for these outcomes. Among patients with Alzheimer's disease randomised to DHA or placebo, the 18-month difference (Δ) in CSF DHA levels between placebo and treatment arms was 54% lower in ε4 carriers than in non-carriers (Δ = 1.0164 in ε4 carriers and Δ = 2.2075 in non-carriers). With an observed SD of 1.027, the effect size (SD of treatment factor means divided by SD) for the DHA treatment main effect was 0.78, and 0.29 for the DHA × APOE ε4 interaction.

The sample size required to detect a 50% lower DHA effect in ε4 carriers compared with non-carriers at 80% power and a two-sided α = 0.05, using a factorial ANOVA with a 1:1 allocation ratio, corresponded to a DHA treatment effect size of 0.75 and an interaction effect size of 0.25, requiring 32 participants per cell (128 total). To account for an anticipated 20% dropout rate, the target sample size was increased to 160 participants (40 per cell). Due to COVID-19-related disruptions, observed dropout during trial conduct approached 30%, and the sample size was increased to 46 participants per cell (184 total) to achieve the planned 128 completers for the lumbar puncture arm.

Additional funding enabled expansion to include a no-lumbar puncture arm (total planned enrolment of 368 participants), providing enhanced power for cognitive outcome analyses. With 30% dropout, the detectable effect size at 80% power is d = 0.35 SD units. In the MIDAS randomised placebo-controlled trial evaluating DHA in adults aged 60 years and older with age-related cognitive decline, the effect size for 24-week change in verbal memory (delayed recall) was 0.3 SD units. Final enrolment was maintained at the expanded sample size with regulatory approval.

### Analysis populations

Three analysis populations were defined prospectively. The intention-to-treat (ITT) population included all randomised participants. The per-protocol (PP) population included participants achieving ≥80% adherence by capsule count. The safety population included all participants who received at least one dose of study product and were followed at least once for adverse events.

### Statistical models

Baseline characteristics were summarised by randomised group using mean (SD) or median (25th, 75th percentiles) for continuous variables and frequency (percent) for categorical variables. Standardised group differences were calculated as a measure of group balance. All outcome analyses followed intention-to-treat principles, including all randomised participants. The estimand for the primary outcome was the mean 6-month change in CSF DHA/AA among participants randomised in the LP arm. Secondary and exploratory outcome estimands were the mean 24-month change in outcome among all randomised participants. The primary analysis used mixed-effects models for repeated measures (MMRM) estimated by restricted maximum likelihood (REML) and a random participant-level intercept; an unstructured covariance matrix and Kenward-Roger denominator degrees-of-freedom adjustment were used. Fixed effects on the primary outcome included randomised treatment, APOE ε4 carrier status, indicator variables for assessment times, clinic strata, and laboratory batch. Analysis of secondary trial outcomes added fixed effects for age, sex, education, ethnicity, and study arm (LP vs no-LP). The time effect estimated the mean change in outcome from baseline; a two-way interaction of treatment x time tested the treatment group differences in the mean change from baseline. Three-way interactions (treatment × time × APOE ε4) tested whether treatment effects varied by genotype.

For the primary outcome measured in participant i at time j (j = baseline or 6 months), the full three-way interaction MMRM model was:

$$y_{\text{ij}}=\beta_{0}+\beta_{1}T_{i}+\beta_{2}{APOE4}_{i}{+\beta }_{3}\;I\left({Time}_{ij}=6\text{mo}\right)+\beta_{4}\left(T_{i}*I\left({Time}_{ij}=6\text{mo}\right)\right)+\beta_{5}\left({APOE4}_{i}*I\left({Time}_{ij}=6\text{mo}\right)\right)+\beta_{6}\left(T_{i}*{APOE4}_{i}\right)+\beta_{7}\left({T_{i}*APOE4}_{i}*I\left({Time}_{ij}=6\text{mo}\right)\right)+\beta_{8}\left({clinic}_{i}\right)+\beta_{9}\left({lab\;batch}_{ij}\right)+\alpha_{i}{+\varepsilon }_{\text{ij}}$$

with indicator variables $T_{i}$ (1 = DHA, 0 = placebo), ${APOE4}_{i}$ (1 = carrier, 0 = non-carrier), ${clinic}_{i}$ (1 = academic centre, 0 = LA County DHS clinic) and bolded parameters represent a vector of indicator variables for lab batch, $\alpha_{i}$ represents the participant-level random intercept and $\varepsilon_{\text{ij}}$ represents the residual error.

In the safety population, adverse events were coded using the MedDRA system and summarised by MedDRA body system categories. Events were summarised by randomised group as participants reporting: any adverse event, serious adverse events, deaths, and adverse events of special interest (AESI) to DHA supplementation. Events were summarised by randomised group numbers and proportions, along with a 95% exact binomial confidence limit on the group differences (DHA vs placebo).

### Missing data

The trial experienced 38% dropout by 24 months, primarily due to COVID-19 pandemic disruptions. MMRM analyses assume data are missing at random (MAR), conditional on observed data and covariates in the model. This assumption was explored by comparing baseline characteristics between completers and non-completers (Supplemental Table S1b). No imputation of missing outcome data was performed.

### Statistical inference

Statistical significance was set at 2-sided p < 0.05 for the primary outcome. For secondary and exploratory outcomes, point estimates and 95% confidence intervals are reported without p-values, emphasising estimation over hypothesis testing consistent with the Phase 2a study design. No adjustment for multiple comparisons was applied to exploratory outcomes, with results interpreted cautiously. All analyses used SAS version 9.4 and R version 4.2.1.

### Role of the funding source

This study was funded by the US National Institute on Aging (R01AG057684) and the Alzheimer's Drug Discovery Foundation (GC-201711-2014197). The funding agencies had no role in study design, data collection, analysis, interpretation, or the decision to submit for publication. The corresponding author had full access to study data and final responsibility for the decision to submit for publication.

## Results

**Fig. 1:**
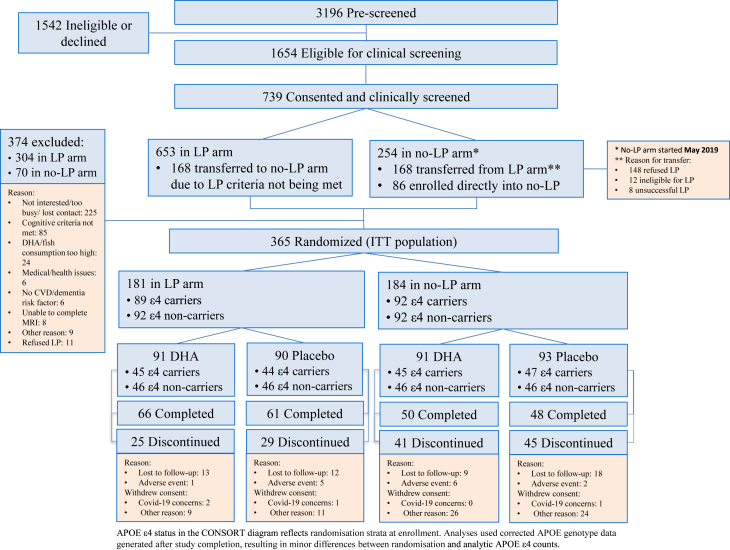
**CONSORT flow diagram for the PreventE4 trial.** CONSORT flow diagram showing screening, randomisation, allocation, follow-up, and analysis of participants in the PreventE4 trial. Reasons for exclusion and discontinuation are shown at each stage. LP, Lumbar Puncture arm; DHA, docosahexaenoic acid; MRI, magnetic resonance imaging; CVD, cardiovascular disease.

Participants were recruited between September 2018 and May 2024. Of 739 individuals screened, 365 were randomised: 181 in the LP arm and 184 in the no-LP arm (Fig. 1). Baseline characteristics were balanced between groups (Table 1). Mean age was 66.4 years, 210 (58%) were female, 142 (39%) were Hispanic, and 173 (47%) were APOE ε4 carriers. Most participants (299 [82%]) identified as white, and 251 (69%) reported low physical activity.

**Table 1:** Baseline demographic, clinical, and laboratory characteristics of all randomised participants (N = 365).

Variable	Placebo *(N = 183)*	DHA *(N = 182)*
Age in years, mean (SD)	66.4 (5.8)	66.4 (5.7)
Sex, n (%)		
Female	113 (62%)	97 (53%)
Male	70 (38%)	85 (47%)
Ethnicity, n (%)		
Not Hispanic	115 (63%)	108 (59%)
Hispanic	68 (37%)	74 (41%)
Race, n (%)		
Asian or Pacific Islander	19 (10%)	11 (6%)
Black or African American	14 (8%)	12 (7%)
Native American	1 (<1%)	1 (<1%)
White	147 (80%)	152 (84%)
Other/mixed race	2 (1%)	6 (3%)
Education <12 years, n (%)	26 (14%)	22 (12%)
Hypertension, n (%)	93 (51%)	95 (52%)
Hyperlipidaemia, n (%)	119 (65%)	129 (71%)
Exercise <3 days/week, n (%)	137 (75%)	115 (63%)
Moderate and vigorous MET hours/week, median (IQR)	5.3 (0.0, 9.0)	4.5 (0.5, 9.0)
DHA intake, mg average per day, median (IQR)	85.0 (51.0, 120.0)	89.0 (58.0, 123.0)
BMI >30 kg/m^2^, n (%)	70 (38%)	71 (39%)
BMI kg/m^2^, mean (SD)	28.9 (5.3)	29.0 (5.4)
Blood pressure, mean (SD)	*(N = 175)*	*(N = 177)*
Systolic blood pressure, mm Hg	135.9 (18.3)	135.5 (17.8)
Diastolic blood pressure, mm Hg	80.1 (10.1)	79.9 (9.6)
Cognitive testing		
FAQ score, median (IQR)	0.0 (0.0, 0.0)	0.0 (0.0, 0.0)
MMSE score, median (IQR)	29.0 (28.0, 30.0) *(N = 119)*	29.0 (28.0, 30.0) *(N = 113)*
MOCA-blind score, median (IQR)	20.0 (18.5, 20.5) *(N = 64)*	20.0 (19.0, 21.0) *(N = 69)*
Weschler Logical Memory II immediate recall score, median (IQR)	12.0 (10.0, 14.0)	12.0 (9.0, 15.0)
Weschler Logical Memory II delayed score, median (IQR)	11.0 (8.0, 13.0)	11.0 (9.0, 14.0)
RBANS total scale index, mean (SD)	94.8 (17.0) *(N = 183)*	94.3 (15.9) *(N = 181)*
CSF Total PUFA, median (IQR)	*(N = 80)*	*(N = 81)*
Total DHA μg/mL	0.24 (0.19, 0.29)	0.22 (0.19, 0.28)
Total DHA/AA ratio	0.46 (0.37, 0.53)	0.43 (0.38, 0.49)
RBC Total PUFA, median (IQR)^a^	*(N = 26)*	*(N = 29)*
Total DHA %	3.78 (3.27, 5.46)	4.30 (3.48, 5.24)
Total DHA/AA ratio	0.23 (0.19, 0.30)	0.26 (0.20, 0.30)
Plasma total PUFA, median (IQR)	*(N = 118)*	*(N = 127)*
Total DHA nmol/mL	287.4 (205.4, 342.7)	271.4 (208.2, 349.1)
Total DHA/AA ratio	0.26 (0.19, 0.33)	0.25 (0.21, 0.34)
Plasma free PUFA, median (IQR)	*(N = 111)*	*(N = 118)*
Free DHA ng/mL	762.8 (551.6, 900.3)	726.8 (547.8, 985.0)
Free DHA/AA ratio	0.87 (0.72, 1.27)	0.90 (0.72, 1.15)

a RBCs were collected in a small subset of participants.

The study had a 38% dropout rate over 24 months, primarily due to COVID-19, with 225 participants completing the trial. Dropouts were more likely to be Hispanic, have lower education levels and baseline RBANS scores, and lower plasma DHA concentrations. Baseline characteristics of trial completers vs dropouts are presented in Supplemental Table S1b. The compliance rate was similar in the placebo and treatment arms (Supplemental Table S1c).

**Fig. 2:**
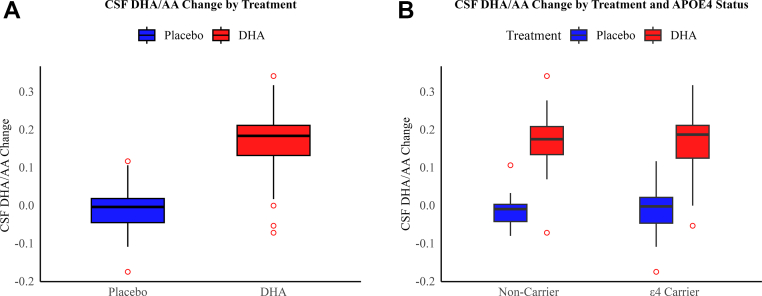
**Change in CSF DHA/AA ratio over 6 months.** (A) DHA treatment (n = 86) increased CSF DHA/AA ratio (mean change = +0.17) compared with placebo (n = 81, mean change = −0.02, p < 0.0001). (B) Similar mean changes were observed in APOE ε4 carriers (+0.17 vs −0.02) and non-carriers (+0.17 vs −0.01), with no genotype interaction. Changes were estimated from linear mixed-effects models adjusted for APOE4 status, clinic site, and laboratory batch, with marginal means and contrasts estimated using *emmeans*. Boxplots show median, IQR, and outliers. CSF, cerebrospinal fluid; DHA, docosahexaenoic acid; AA, arachidonic acid.

Among 167 participants with CSF data (DHA: n = 86; placebo: n = 81, Supplemental Table S1c), DHA supplementation significantly increased CSF DHA/AA ratio at 6 months compared with placebo (mean change: DHA 0.17 [95% CI 0.15–0.18] vs placebo −0.02 [95% CI −0.04 to −0.0004]; difference 0.19 [95% CI 0.16–0.21]; p < 0.0001; Cohen's d = 1.64). The effect was consistent regardless of APOE ε4 status (treatment × APOE ε4 interaction p = 0.71) (Fig. 2, Supplemental Table S2).

**Fig. 3:**
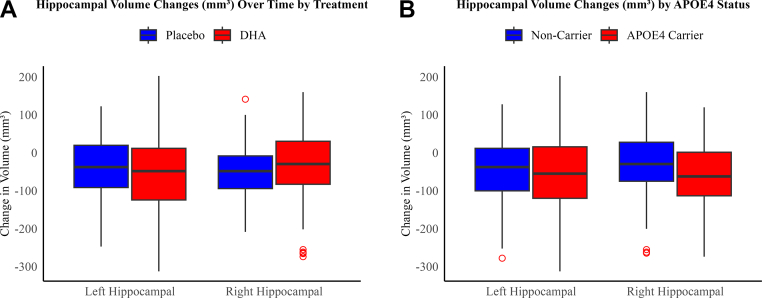
**Change in hippocampal volume over 24 months.** (A) No significant difference in hippocampal volume change was observed between DHA and placebo groups in the left hippocampus (–15.69 [n = 170] vs –7.92 [n = 177]) or the right hippocampus (–3.48 [n = 172] vs –14.24 [n = 175]). (B) No differences were observed when participants were stratified by APOE4 carrier status. Estimates were derived from linear mixed-effects models adjusted for treatment, APOE4 status, clinic strata, study arm, age, sex, and mean intracranial volume. Boxplots show median, IQR, and outliers. DHA, docosahexaenoic acid; IQR, interquartile range; ICV, intracranial volume.

Among 347 participants with hippocampal volume (HV) data (left: DHA n = 170, placebo n = 177; right: DHA n = 172, placebo n = 175), HV at 24 months declined modestly in both hemispheres. For the left hippocampal volume (HV), annual percent decline was slightly greater in the DHA group (−0.75%, SD 1.57; n = 97) than placebo (−0.63%, SD 1.21; n = 96), and greater in APOE4 carriers (−0.77%, SD 1.54; n = 88) than non-carriers (−0.62%, SD 1.27; n = 105). For the right HV, decline was slightly less in the DHA group (−0.55%, SD 1.35; n = 97) compared with placebo (−0.67%, SD 1.06; n = 90), while APOE4 carriers again showed greater decline (−0.83%, SD 1.21; n = 84) than non-carriers (−0.42%, SD 1.20; n = 103). DHA supplementation had no significant effect on HV changes (mean group differences in change: left −7.77 [95% CI −33.83 to 18.29] mm^3^; right 10.75 [−13.75 to 35.26] mm^3^) (Fig. 3, Supplemental Table S3).

Among the 364 participants who completed cognitive assessments, 181 were in the DHA group and 183 were in the placebo group. After 24 months, *APOE* ε4 non-carriers experienced a greater improvement in Total RBANS scores than carriers (non-carrier:+3.79 [95% CI: 2.18–5.40]; carrier: +1.60 [95% CI: −0.03 to 3.24]; mean group difference: 2.19 [95% CI −0.11 to 4.48]) (Supplemental Figure S1). DHA treatment did not influence Total RBANS scores at 24 months, mean change was +2.76 [95% CI: 1.15–4.37] in the DHA group vs +2.67 [95% CI: 1.02–4.32] in the placebo group (mean group difference: 0.09 [95% CI −2.21 to 2.39]). *APOE* status did not modify the treatment response on Total RBANS (Supplemental Table S4).

DHA supplementation increased red blood cell DHA/AA ratio at 24 months (DHA: +0.46 [95% CI 0.40–0.51] vs placebo: +0.01 [95% CI −0.05 to 0.07]) and omega-3 index (DHA: 6.18% [95% CI 5.46–6.90] vs placebo: 0.21% [95% CI −0.54 to 0.96]). Plasma DHA/AA also increased at 6 months. Treatment effects were consistent between APOE ε4 carriers and non-carriers (Supplemental Tables S5, S6a and b and Supplemental Figures S2, S3a and b).

Safety outcomes were assessed in all participants who received at least one dose of study product (DHA: n = 181; placebo: n = 181). Adverse events occurred in 73 (40.3%) of DHA participants and 74 (40.9%) of placebo participants (risk difference: −0.6% [95% CI −10.7% to 9.6%]). Serious adverse events occurred in 5 (2.8%) DHA participants and 13 (7.2%) placebo participants (risk difference: −4.4% [95% CI −9.5% to 0.1%]). Two deaths occurred (one per group), neither attributed to study intervention. No adverse events of special interest differed between groups (Table 2).

**Table 2:** Table of adverse events among the study population (N = 362).

	Placebo, N = 181	DHA, N = 181
	N (%)^a^	N (%)^a^
**Adverse events**	74 (40.9)	73 (40.3)
**AEs of Special Interest (AESI)**	14 (7.7)	8 (4.4)
Diarrhoea	6	3
Eructation	2	0
Faeces soft	1	0
Flatulence	1	0
Frequent bowel movements	1	0
Nausea	4	0
Contusion	1	0
Back pain, post-LP	0	1
Headache, post-LP	2	4
**Non-AESI**	69 (38.1)	69 (38.1)
**Serious adverse events**	13 (7.2)	5 (2.8)
Atrial fibrillation	0	1
Cardiomyopathy	1	0
Large intestine perforation	0	1
COVID-19 (death)	0	1
Pneumonia	2	0
Subcutaneous abscess	1	0
Bladder neoplasm	1	0
Breast cancer	2	1
Endometrial cancer	0	1
Gastric cancer	1	0
Lung cancer	1	0
Ovarian cancer	1	0
Throat cancer	1	0
Cerebrovascular accident (death)	1	0
Colectomy	1	0
Coronary artery bypass	1	0
Hospitalisation (descending aorta)	1	0
Knee operation	0	1
**Deaths**	1 (0.6)	1 (0.6)

a Number of participants experiencing an event and % of total number of participants, by treatment group; for rows with specific events, the total number of events by treatment group are shown.

## Discussion

This phase 2a trial achieved its primary objective: high-dose DHA supplementation (2 g/day) robustly increased CSF DHA/AA within 6 months independent of APOE ε4 genotype. This establishes that brain DHA delivery is achievable in cognitively unimpaired older adults with low omega-3 intake, regardless of APOE ε4 status. However, confirmed CNS target engagement did not translate to benefits in hippocampal volume, cortical thickness, or cognitive performance over 24 months. This contradicts our initial hypothesis that APOE ε4 carriers would show impaired brain DHA delivery before dementia, unlike the dysregulated DHA blood-CSF transport observed in APOE ε4 carriers with established dementia.^18^^,^^19^

These findings address a critical uncertainty that has limited interpretation of prior omega-3 prevention trials. Earlier studies using lower DHA doses (≤1 g/day) showed no cognitive benefit,^12^ but it remained unclear whether null results reflected insufficient brain delivery, inadequate dosing, or true biological inefficacy. By directly measuring CSF DHA levels, we demonstrate that high-dose supplementation achieves robust CNS target engagement even in individuals with chronically low omega-3 intake. The large effect size for CSF DHA enrichment (Cohen's d = 1.6) confirms that brain delivery is not the limiting factor. This distinguishes our trial from prior studies where brain penetration could not be verified. The disconnect between confirmed biochemical target engagement and absent clinical benefit over 24 months indicates that brain omega-3 enrichment alone is insufficient for cognitive preservation in this population and timeframe. This finding redirects the field away from dose-escalation or dose formulation strategies and toward understanding mechanisms of brain DHA metabolism, which populations, intervention durations, or combination approaches might yield measurable clinical benefits. The failure of high-dose DHA supplementation to modify clinical outcomes despite confirmed CNS delivery suggests that membrane phospholipid composition changes alone are insufficient. Pathological activation of enzymes such as calcium-dependent phospholipase A2 (cPLA2)^20^ may actively alter DHA and AA fates after supplementation, catabolizing these polyunsaturated fatty acids. This process attenuates the intended biochemical intervention at the subcellular compartments (synaptic membranes) most critical for cognition.

Our results align with the broader pattern of null findings in omega-3 supplementation trials in cognitively unimpaired populations. The VITAL study found no cognitive benefits from omega-3 supplementation (1 g/day EPA + DHA) over 2–3 years in 4218 healthy older adults.^21^ Similarly, systematic reviews indicate that only 21% of omega-3 trials in cognitively normal populations show positive effects.^12^ The LipiDiDiet trial, testing a multinutrient intervention including DHA in prodromal AD patients, did not meet its prespecified primary cognitive endpoint at 24 months.^22^ This null finding at 24 months occurred despite testing in a more impaired population (prodromal AD) who might be expected to show greater treatment responsiveness than our cognitively unimpaired cohort. The multinutrient formulation in LipiDiDiet (containing DHA, EPA, and multiple other nutrients) also differed from our DHA monotherapy approach.

### Lessons learnt

Our findings reveal a disconnect between achieving brain DHA target engagement and clinical benefit, with important implications for prevention trial design. Despite robust DHA brain delivery, this relatively healthy population (minimal baseline decline, high education) with concurrent vascular risk factors (69% physically inactive, 39% obese, 52% hypertensive) showed no cognitive or structural benefits over 24 months. The inflammatory states promoted by concurrent vascular and metabolic risk factors^16^ may limit single-nutrient efficacy of supplements like DHA. DHA supplementation as monotherapy may be insufficient when multiple pathophysiological processes drive neurodegeneration and alter the fate of DHA in the brain. Multimodal interventions that simultaneously address physical inactivity, vascular health, and other modifiable risk factors, such as the FINGER^23^ and SMARRT^24^ trials are likely more successful in improving brain DHA metabolism.

Alternatively, future trials may benefit from targeting individuals with biomarker evidence of early neurodegeneration rather than purely asymptomatic individuals, as this population may be more likely to demonstrate measurable decline and treatment response over feasible trial durations. Standard cognitive and structural MRI measures may lack sensitivity in early-stage, asymptomatic populations. Incorporating more sensitive markers of neurodegeneration, including plasma phosphorylated tau, neurofilament light chain, advanced imaging markers (e.g., DHA PET^13^), or detailed neuropsychological assessments targeting subtle executive function changes could improve the detection of treatment effects. Beyond APOE ε4 status, information from changes in the gut microbiome^25^ may identify subgroups most likely to benefit from omega-3 supplementation. While 24-month trials are feasible and commonly used, the stability of outcomes in healthy populations over this period may necessitate either longer trials (36 months or longer as suggested by LipiDiDiet extended follow-up^26^).

This trial's strengths include recruitment of an ethnically diverse, APOE ε4-enriched population with low baseline omega-3 intake; biochemical confirmation of target engagement; comprehensive outcome assessments; and rigorous trial methodology. The study addresses a clinical question using appropriate population and intervention characteristics. However, several limitations affect interpretation. Our study population demonstrated preserved cognitive and structural brain integrity, with minimal hippocampal atrophy (<0.5% annually) and improved cognitive scores in both groups. This preservation likely reflects the relatively young age (mean 66 years), high education levels, and early disease stage despite enriching for vascular risk factors, potentially limiting detectable treatment effects in this population over the study timeframe. The relatively young, highly educated population with minimal baseline cognitive or structural brain changes may not represent typical clinical prevention populations. The single-centre site limits generalisability with the 38% dropout rate, primarily due to COVID-19, potentially biased results and reduced statistical power for clinical outcomes. Participants who withdrew differed from completers, possibly also affecting generalisability. Additionally, the study's focus on a single nutrient may have limited efficacy compared with more comprehensive interventions addressing multiple pathways simultaneously. Finally, questionnaire-based lifestyle measures with broad cognitive inclusion criteria increase measurement variability.

By demonstrating robust CNS target engagement in an APOE ε4–enriched cohort with low baseline omega-3 intake, this trial establishes that high-dose DHA supplementation is safe, achievable, and informative, while clarifying that broad supplementation alone is insufficient to modify cognitive or structural outcomes in a relatively healthy population over 2 years. Building on these findings, omega-3 metabolism remains mechanistically relevant in APOE ε4 carriers. Future work should shift from uniform supplementation toward mechanistic studies of brain DHA metabolism and personalised multimodal strategies that integrate genotype, lipid and inflammatory biomarkers, and more sensitive CNS endpoints to identify the subgroups most likely to benefit.

## Contributors

HNY designed and supervised the trial, obtained funding and wrote the manuscript, SGP helped with data analysis and drafting the manuscript, NA assisted with drafting the manuscript, MJ helped with cognitive testing, ICA, DD coordinated study visits, AS assisted blood sample collection and storage, BK, MVD and IA assisted with biomarker measurements, SL supervised biomarker management, JP assisted with study coordination, NK led data management, LD supervised cognitive testing, HC helped with study design, WJM supervised all data analysis and design, MGH performed lumbar punctures, MNB oversaw the imaging biomarkers, LSS supervised study design, grant applications and writing the manuscript. NK and WJM accessed and verified the underlying data. All authors read and approved the final version of the manuscript.

## Data sharing statement

De-identified participant data collected for the study, including individual participant data and data dictionaries, will be made available upon reasonable request to the corresponding author beginning 6 months after publication and ending 10 years after publication. Data will be shared with researchers whose proposed use of the data has been approved by an independent review committee, for the purposes of achieving the aims in the approved proposal. Data will be shared under a data access agreement. Requests should be directed to the corresponding author.

## Declaration of interests

The authors declare that they have no conflicts of interest. LSS reports grants and contracts from NIH and industry partners (including Biogen, Eisai, Eli Lilly, Roche/Genentech, and others), and consulting fees from multiple organisations including AC Immune, Cortexyme, Lundbeck, Merck, and Novo Nordisk, outside the submitted work. All other authors declare no competing interests.

## Appendix A Supplementary data

Primary Supplements

Protocol
