# Supplementary material for: CNS target engagement of high-dose DHA supplementation in older adults at risk for dementia: a randomised, double-blind, placebo-controlled trial
Source: eBioMedicine. 2026 Jun 18;129:106316. doi: 10.1016/j.ebiom.2026.106316 (PMC13347776; doi:10.1016/j.ebiom.2026.106316)
Supplement: Primary Supplements [file mmc1.docx]

**CNS Target Engagement of High-dose DHA Supplementation in Older Adults at Risk for Dementia: A Randomised, Double-blind, Placebo-controlled Trial**

Hussein N. Yassine^1,3^, Sara Ghasem Pour^1^, Marlene Juarez^1^, Isabella C. Arrelanas^1^, Nada Ali^1^, Dante Dikeman^2^, Ashley Sanchez^1^, Jackson Park^1^, Bilal Kerman^3^, Marlon V. Duro^4^, Isaac Asante^1,5^, Stan Louie^2^, Naoko Kono^6^, Lina D’Orazio^1^, Helena Chui^1^, Wendy J. Mack^6^, Michael G. Harrington^1^, Meredith N. Braskie^7^, and Lon S. Schneider^1^

**^1^Department of Neurology, Keck School of Medicine, University of Southern California, Los Angeles, CA, United States** (H N Yassine MD, S G Pour, M Juarez, I C Arrelanas, N Ali, A Sanchez, I Asante PhD, J Park, Lina D’Orazio^,^ PhD, H Chui MD, M G Harrington MB ChB, L S Schneider MD)

**^2^Department of Clinical Pharmacy, Alfred E. Mann School of Pharmacy and Pharmaceutical Sciences, University of Southern California, Los Angeles, CA, United States** (D Dikeman, S Louie PhD)

**^3^Department of Medicine, University of Southern California, Los Angeles, CA, United States** (H N Yassine MD, B Kerman PhD)

**^4^Department of Radiology, Keck School of Medicine, University of Southern California, Los Angeles, CA, United States** (M V Duro PhD)

**^5^Institute for Technology and Medical Systems (ITEMS), University of Southern California, Los Angeles, CA, United States** (I Asante PhD)

**^6^Department of Population and Public Health Sciences, University of Southern California, Los Angeles, CA, United States** (N Kono, W J Mack PhD)

**^7^Mark and Mary Stevens Neuroimaging and Informatics Institute, University of Southern California, Los Angeles, CA, United States** (M N Braskie PhD)

Correspondence to:

Dr. Hussein N. Yassine,

Department of Neurology, University of Southern California, Los Angeles, 90033, United States

[**hyassine@usc.edu**](mailto:hyassine@usc.edu)

**Table of Contents**

1. **Supplemental Tables**
   1. Supplemental Table 1a. Baseline Demographic, Clinical, and Laboratory Characteristics of Randomized Participants by Treatment Group and APOE4 Carrier Status
   2. Supplemental Table 1b. Baseline Demographic, Clinical, and Laboratory Characteristics of Trial Completers vs. Dropouts
   3. Supplemental Table 1c. Compliance Rate by Treatment Arm
   4. Supplemental Table 1d. Baseline Demographic, Clinical, and Laboratory Characteristics of Randomized Participants in the Primary Outcome Analysis
   5. Supplemental Table 2. CSF DHA/AA Change at 6 Months
   6. Supplemental Table 3. Treatment Effect on Hippocampal Volume at 24 Months
   7. Supplemental Table 4. Treatment Effect on RBANS Scores at 24 Months
   8. Supplemental Table 5. Plasma DHA/AA Change at 6 Months
   9. Supplemental Table 6a. RBC DHA/AA Change at 24 Months
   10. Supplemental Table 6b. RBC Omega-3 Index (%) Change at 24 Months
2. **Supplemental Figures**

2.1. Supplemental Figure 1. Change in RBANS Composite Score Over 24 Months by Treatment and APOE4
2.2. Supplemental Figure 2. Intervention Increases Plasma DHA/AA Independent of APOE4
2.3. Supplemental Figure 3a. Intervention Increases RBC DHA/AA Independent of APOE4
2.4. Supplemental Figure 3b. Change in RBC Omega-3 Index (%) by Treatment Group and APOE4

| Supplemental Table 1a. Baseline Demographic, Clinical, and Laboratory Characteristics of all Randomized Participants by Treatment Group and APOE4 Carrier Status^1^ | | | | |
| --- | --- | --- | --- | --- |
|  | **Placebo** | | **DHA** | |
| Variable | **No E4**  ***(N=100)*** | **E4 carrier**  ***(N=83)*** | **No E4**  ***(N=92)*** | **E4 carrier**  ***(N=90)*** |
| Age in years, mean (SD) | 66.5 (5.6) | 66.3 (6.0) | 67.0 (5.6) | 65.8 (5.9) |
| Gender, n (%) |  |  |  |  |
| Female | 57 (57%) | 56 (67%) | 45 (49%) | 52 (58%) |
| Male | 43 (43%) | 27 (33%) | 47 (51%) | 38 (42%) |
| Ethnicity, n (%) |  |  |  |  |
| Not Hispanic | 57 (57%) | 58 (70%) | 48 (52%) | 60 (67%) |
| Hispanic | 43 (43%) | 25 (30%) | 44 (48%) | 30 (33%) |
| Race, n (%) |  |  |  |  |
| Asian or Pacific Islander | 15 (15%) | 4 (5%) | 8 (9%) | 3 (3%) |
| Black or African American | 7 (7%) | 7 (8%) | 4 (4%) | 8 (9%) |
| Native American | 1 (1%) | 0 | 0 | 1 (1%) |
| White | 75 (75%) | 72 (87%) | 76 (83%) | 76 (84%) |
| Other/mixed race | 2 (2%) | 0 | 4 (4%) | 2 (2%) |
| Education <12 years, n (%) | 15 (15%) | 11 (13%) | 11 (12%) | 11 (12%) |
| Hypertension, n (%) | 52 (52%) | 41 (49%) | 54 (59%) | 41 (46%) |
| Hyperlipidemia, n (%) | 64 (64%) | 55 (66%) | 70 (76%) | 59 (66%) |
| Exercise <3 days/week, n (%) | 68 (68%) | 69 (83%) | 60 (65%) | 55 (61%) |
| Moderate and vigorous MET hours/week, median (IQR) | 6.0 (0.0, 9.0) | 4.8 (0.0, 9.0) | 4.5 (0.0, 8.8) | 6.0 (2.3, 10.5) |
| DHA intake, mg average per day, median (IQR) | 85.0 (51.5, 117.0) | 87.0 (50.0, 122.0) | 84.5 (57.3, 107.0) | 94.5 (59.0, 143.0) |
| BMI >30 kg/m^2^, n (%) | 36 (36%) | 34 (41%) | 39 (42%) | 32 (36%) |
| BMI kg/m^2^, mean (SD) | 28.8 (5.1) | 28.9 (5.5) | 29.4 (5.0) | 28.6 (5.7) |
| Blood pressure, mean (SD) | *(N=97)* | *(N=78)* | *(N=91)* | *(N=86)* |
| Systolic blood pressure, mm Hg | 137.7 (20.0) | 133.6 (15.6) | 137.8 (15.8) | 133.1 (19.4) |
| Diastolic blood pressure, mm Hg | 80.9 (10.1) | 79.0 (10.2) | 81.0 (8.7) | 78.8 (10.4) |
| Cognitive testing |  |  |  |  |
| FAQ score, median (IQR) | 0.0, (0.0, 0.0) | 0.0, (0.0, 0.0) | 0.0, (0.0, 0.0) | 0.0, (0.0, 0.0) |
| MMSE score, median (IQR) | 29.0 (27.0, 30.0)  *(N=80)* | 29.0 (28.0, 30.0)  *(N=39)* | 29.0 (28.0, 30.0)  *(N=72)* | 29.0 (28.0, 30.0)  *(N=41)* |
| MOCA-blind score, median (IQR) | 19.5 (18.5, 20.0)  *(N=20)* | 20.0 (18.5, 21.0)  *(N=44)* | 20.0 (18.5, 20.5)  *(N=20)* | 20.0 (19.0, 21.0)  *(N=49)* |
| Weschler Logical Memory II immediate recall score, median (IQR) | 12.0 (10.0, 14.0) | 12.0 (9.0, 14.0) | 12.0 (9.0, 14.0) | 13.0 (9.0, 15.0) |
| Weschler Logical Memory II delayed score, median (IQR) | 10.5 (8.0, 13.0) | 11.0 (8.0, 13.0) | 11.0 (9.0, 13.5) | 11.0 (9.0, 14.0) |
| RBANS total scale index, mean (SD) | 93.4 (18.8) | 96.6 (14.4) | 92.2 (16.0)  *(N=91)* | 96.4 (15.7) |
| CSF Total PUFA, median (IQR) | *(N=44)* | *(N=36)* | *(N=45)* | *(N=36)* |
| Total DHA ug/mL | 0.24 (0.18, 0.28) | 0.24 (0.19, 0.30) | 0.23 (0.19, 0.28) | 0.22 (0.19, 0.26) |
| Total DHA/AA ratio | 0.48 (0.38, 0.54) | 0.43 (0.36, 0.50) | 0.43 (0.38, 0.50) | 0.45 (0.38, 0.48) |
| RBC Total PUFA, median (IQR) | *(N=14)* | *(N=12)* | *(N=13)* | *(N=16)* |
| Total DHA % | 4.94 (3.47, 5.70) | 3.33 (3.02, 3.98) | 4.36 (3.62, 5.24) | 3.99 (3.31, 5.29) |
| Total DHA/AA ratio | 0.28 (0.21, 0.36) | 0.19 (0.18, 0.23) | 0.28 (0.21, 0.33) | 0.22 (0.19, 0.29) |
| Plasma Total PUFA, median (IQR) | *(N=67)* | *(N=51)* | *(N=62)* | *(N=65)* |
| Total DHA nmol/mL | 259.1 (196.4, 342.7) | 305.3 (221.4, 351.5) | 257.9 (203.4, 328.4) | 284.9 (217.4, 380.2) |
| Total DHA/AA ratio | 0.25 (0.19, 0.33) | 0.26 (0.20, 0.33) | 0.25 (0.19, 0.34) | 0.25 (0.22, 0.34) |
| Plasma Free PUFA, median (IQR) | *(N=67)* | *(N=44)* | *(N=59)* | *(N=59)* |
| Free DHA ng/mL | 788.2 (523.5, 924.9) | 695.8 (551.6, 860.3) | 730.4 (548.8, 1033.6) | 688.3 (544.1, 878.6) |
| Free DHA/AA ratio | 0.87 (0.72, 1.15) | 0.92 (0.68, 1.29) | 0.92 (0.72, 1.18) | 0.88 (0.72, 1.12) |

^1^ E4 carrier status based on the full APOE genotypes determined at the end of the study.

| Supplemental Table 1b. Baseline Demographic, Clinical, and Laboratory Characteristics of Trial Completers vs. Dropouts | | |
| --- | --- | --- |
| Variable | **Completers**  **(N=225)** | **Dropouts**  **(N=140)** |
| Age in years, mean (SD) | 66.5 (5.7) | 66.3 (5.9) |
| Gender, n (%) |  |  |
| Female | 126 (56%) | 84 (60%) |
| Male | 99 (44%) | 56 (40%) |
| Ethnicity, n (%) |  |  |
| Not Hispanic | 154 (68%) | 69 (49%) |
| Hispanic | 71 (32%) | 71 (51%) |
| Race, n (%) |  |  |
| Asian or Pacific Islander | 22 (10%) | 8 (6%) |
| Black or African American | 15 (7%) | 11 (8%) |
| Native American | 0 | 2 (1%) |
| White | 184 (82%) | 115 (82%) |
| Other/mixed | 4 (2%) | 4 (3%) |
| Education <12 years, n (%) | 19 (8%) | 29 (21%) |
| Hypertension (yes), n (%) | 111 (49%) | 77 (55%) |
| Hyperlipidemia (yes), n (%) | 160 (71%) | 88 (63%) |
| Exercise <3 days/week, n (%) | 149 (66%) | 103 (74%) |
| Moderate and vigorous MET hours/week, median (IQR) | 6.0 (0.0, 9.0) | 4.8 (0.0, 8.3) |
| DHA intake, mg average per day, median (IQR) | 91.0 (61.0, 121.0) | 76.0 (44.5, 121.5) |
| BMI >30 kg/m^2^, n (%) | 85 (38%) | 56 (40%) |
| BMI kg/m^2^, mean (SD) | 28.8 (5.1) | 29.2 (5.7) |
| Blood pressure, mean (SD) | *(N=216)* | *(N=136)* |
| Systolic blood pressure, mm Hg | 134.9 (18.4) | 137.0 (17.2) |
| Diastolic blood pressure, mm Hg | 79.7 (9.5) | 80.5 (10.4) |
| Cognitive testing |  |  |
| FAQ score, median (IQR) | 0.0 (0.0, 0.0) | 0.0 (0.0, 1.0) |
| MMSE score, median (IQR) | 29.0 (28.0, 30.0)  *(N=140)* | 29.0 (27.0, 30.0)  *(N=92)* |
| MOCA-blind score, median (IQR) | 20.0 (19.0, 20.0)  *(N=85)* | 19.0 (18.0, 21.0)  *(N=48)* |
| Weschler Logical Memory II immediate recall score, median (IQR) | 12.0 (10.0, 15.0) | 12.0 (9.0, 14.0) |
| Weschler Logical Memory II delayed score, median (IQR) | 11.0 (9.0, 13.0) | 10.0 (8.0, 13.0) |
| RBANS total scale index, mean (SD) | 98.3 (15.7) | 88.6 (15.9)  *(N=139)* |
| CSF Total PUFA, median (IQR) | *(N=116)* | *(N=45)* |
| Total DHA ug/mL | 0.22 (0.18, 0.27) | 0.24 (0.20, 0.30) |
| Total DHA/AA ratio | 0.43 (0.37, 0.50) | 0.47 (0.41, 0.54) |
| RBC Total PUFA, median (IQR) | *(N=53)* | *(N=2)* |
| Total DHA % | 4.04 (3.29, 5.09) | 5.66 (5.27, 5.76) |
| Total DHA/AA ratio | 0.24 (0.19, 0.29) | 0.39 (0.33, 0.44) |
| Plasma Total PUFA, median (IQR) | *(N=206)* | *(N=39)* |
| Total DHA nmol/mL | 283.9 (211.5, 349.1) | 252.9 (176.5, 334.1) |
| Total DHA/AA ratio | 0.25 (0.20, 0.33) | 0.24 (0.19, 0.34) |
| Plasma Free PUFA, median (IQR) | *(N=205)* | *(N=24)* |
| Free DHA ng/mL | 765.9 (563.1, 950.9) | 548.6 (414.2, 646.2) |
| Free DHA/AA ratio | 0.89 (0.72, 1.21) | 0.85 (0.72, 1.06) |

| **Supplemental 1c.** Overall median study adherence(%) by treatment | | |
| --- | --- | --- |
| **Treatment** | **N** | **Median (IQR)** |
| Placebo | 128 | 84.8 (76.0, 92.8) |
| DHA | 144 | 88.0 (79.7, 94.2) |

| Supplemental Table 1d. Baseline Demographic, Clinical, and Laboratory Characteristics of Participants in the Primary Outcome Analysis (N=167) | | |
| --- | --- | --- |
| Variable | **Placebo**  ***(N=81)*** | **DHA**  ***(N=86)*** |
| Age in years, mean (SD) | 66.6 (5.8) | 66.6 (5.4) |
| Gender, n (%) |  |  |
| Female | 55 (68%) | 45 (52%) |
| Male | 26 (32%) | 41 (48%) |
| Ethnicity, n (%) |  |  |
| Not Hispanic | 53 (65%) | 57 (66%) |
| Hispanic | 28 (35%) | 29 (34%) |
| Race, n (%) |  |  |
| Asian or Pacific Islander | 7 (9%) | 5 (6%) |
| Black or African American | 6 (7%) | 6 (7%) |
| Native American | 1 (1%) | 0 (0%) |
| White | 66 (81%) | 71 (83%) |
| Other/mixed race | 1 (1%) | 4 (5%) |
| Education <12 years, n (%) | 10 (12%) | 8 (9%) |
| Hypertension, n (%) | 37 (46%) | 49 (57%) |
| Hyperlipidemia, n (%) | 50 (62%) | 60 (70%) |
| Exercise <3 days/week, n (%) | 60 (74%) | 45 (52%) |
| Moderate and vigorous MET hours/week, median (IQR) | 5.3 (0.0, 9.0) | 6.0 (0.0, 10.0) |
| DHA intake, mg average per day, median (IQR) | 90.0 (60.0, 122.0) | 80.0 (48.0, 111.0) |
| BMI >30 kg/m^2^, n (%) | 30 (37%) | 32 (37%) |
| BMI kg/m^2^, mean (SD) | 29.0 (5.7) | 29.0 (5.3) |
| Blood pressure, mean (SD) |  |  |
| Systolic blood pressure, mm Hg | 136.7 (17.6) | 136.2 (18.9) |
| Diastolic blood pressure, mm Hg | 79.0 (8.5) | 79.5 (10.0) |
| Cognitive testing |  |  |
| FAQ score, median (IQR) | 0.0 (0.0, 0.0) | 0.0 (0.0, 0.0) |
| MMSE score, median (IQR) | 29.0 (28.0, 30.0)  *(N=56)* | 29.0 (28.0, 30.0)  *(N=58)* |
| MOCA-blind score, median (IQR) | 20.0 (19.0, 21.0)  *(N=25)* | 20.0 (19.0, 20.0)  *(N=28)* |
| Weschler Logical Memory II immediate recall score, median (IQR) | 12.0 (11.0, 15.0) | 12.5 (10.0, 15.0) |
| Weschler Logical Memory II delayed score, median (IQR) | 12.0 (9.0, 14.0) | 11.5 (9.0, 14.0) |
| RBANS total scale index, mean (SD) | 98.6 (17.2) | 94.3 (15.7) |
| CSF Total PUFA, median (IQR) | *(N=80)* | *(N=81)* |
| Total DHA ug/mL | 0.24 (0.19, 0.29) | 0.22 (0.19, 0.28) |
| Total DHA/AA ratio | 0.46 (0.37, 0.53) | 0.43 (0.38, 0.49) |
| RBC Total PUFA, median (IQR)^1^ | *(N=20)* | *(N=25)* |
| Total DHA % | 3.53 (3.02, 4.79) | 4.18 (3.42,4.70) |
| Total DHA/AA ratio | 0.22 (0.18, 0.27) | 0.25 (0.19, 0.28) |
| Plasma Total PUFA, median (IQR) | *(N=65)* | *(N=69)* |
| Total DHA nmol/mL | 288.7  (210.4, 338.1) | 269.9  (200.1, 318.0) |
| Total DHA/AA ratio | 0.25 (0.20, 0.34) | 0.24 (0.20, 0.33) |
| Plasma Free PUFA, median (IQR) | *(N=63)* | *(N=66)* |
| Free DHA ng/mL | 710.5 (497.7, 881.3) | 677.5 (524.4, 985.0) |
| Free DHA/AA ratio | 0.85 (0.67, 1.27) | 0.91 (0.74, 1.16) |

| Supplemental Table 2.  CSF DHA/AA Change at 6 Months^1^ | | | | | |
| --- | --- | --- | --- | --- | --- |
| Group | **Sample N^2^** | **Mean change (95% CI)^3^** | **Effect size (d)^4^** | **P-value** | **P-value for APOE** ε4 **interaction** |
| Treatment effect | |  | 1.64 | <.0001^5^ |  |
| DHA | 86 | 0.17 (0.15, 0.18) |  |  |  |
| Placebo | 81 | -0.02 (-0.04, -0.0004) |  |  |  |
| APOE ε4 effect |  |  | -0.07 | 0.72^6^ |  |
| ε4 carrier | 77 | 0.07 (0.04 , 0.10) |  |  |  |
| Non-carrier | 90 | 0.08 (0.05, 0.10) |  |  |  |
| Treatment effect by APOE ε4 | | | 0.08 |  | 0.71 |
| ε4 carrier |  |  | 1.69 | <.0001^7^ |  |
| DHA | 40 | 0.17 (0.14, 0.19) |  |  |  |
| Placebo | 37 | -0.02 (-0.05, 0.004) |  |  |  |
| Non-carrier |  |  | 1.60 | <.0001^7^ |  |
| DHA | 46 | 0.17 (0.14, 0.19) |  |  |  |
| Placebo | 44 | -0.01 (-0.04, 0.009) |  |  |  |

^1^ Median follow-up was 7.3 months (range, 4.8 to 13.9).

^2^ In the placebo group, 80 baseline measures and 58 6-month follow-up measures; in the DHA group, 81 baseline measures and 61 6-month follow-up measures.

^3^ Estimates were derived using mixed-effects models for repeated measures (MMRM); two-way interactions tested for treatment effect (treatment x time) and APOE ε4 effect (APOE ε4 x time); three-way interactions (treatment x time x APOE ε4) tested whether treatment effects varied by APOE ε4; additional covariates were clinic strata and laboratory batch.

^4^ Cohen’s d = group difference in change / standard deviation of change

^5^ P value for the difference in change between DHA and placebo.

^6^ P value for the difference in change between APOE ε4 carrier and non-carrier.

^7^ P value for the difference in change between treatment groups within APOE ε4 strata.

***Change in hippocampal volume after the intervention:***

| **Supplemental Table 3. Treatment Effect on Hippocampal Volume at 24 Months** | | | | |
| --- | --- | --- | --- | --- |
| **Outcome, effect** | **Group** | **Sample size^1^** | **Mean (SD) at baseline** | **Mean change (95% CI)^2^** |
| **Whole Hippocampus (mm^3^) Left** |  |  |  |  |
| **Treatment effect** | **DHA** | 170 | 3192.33 (362.35) | -15.69 (-38.13, 6.75) |
|  | **Placebo** | 177 | 3228.53 (342.98) | -7.92 (-30.38, 14.54) |
| APOE ε4 effect | ε4**carrier** | 161 | 3234.11 (359.99) | -15.95 (-39.13, 7.24) |
|  | **Non-carrier** | 186 | 3190.84 (345.70) | -8.35 (-30.17, 13.46) |
| **Treatment effect by APOE4 effect** |  |  |  |  |
| ε4**carrier** | **DHA** | 81 | 3227.47 (384.73) | -23.98 (-53.76, 5.80) |
|  | **Placebo** | 80 | 3240.74 (335.74) | -7.35 (-38.13, 23.43) |
| **Non-carrier** | **DHA** | 89 | 3160.73 (340.07) | -8.36 (-36.84, 20.12) |
|  | **Placebo** | 97 | 3218.46 (350.26) | -8.50 (-36.37, 19.36) |
| **Whole Hippocampus (mm^3^) Right** | |  |  |  |
| **Treatment effect** | **DHA** | 172 | 3313.01 (365.45) | -3.48 (-24.74, 17.77) |
|  | **Placebo** | 175 | 3359.57 (351.75) | -14.24 (-36.02, 7.55) |
| APOE ε4 effect | ε4**carrier** | 161 | 3355.58 (372.28) | -24.23 (-46.34, -2.12) |
|  | **Non-carrier** | 186 | 3320.38 (347.28) | 3.98 (-16.73, 24.69) |
| **Treatment effect by** APOE ε4 | |  |  |  |
| ε4**carrier** | **DHA** | 82 | 3350.25 (376.42) | -25.30 (-52.85, 2.24) |
|  | **Placebo** | 79 | 3361.12 (370.32) | -23.19 (-52.88, 6.50) |
| **Non-carrier** | **DHA** | 90 | 3279.91 (354.22) | 15.92 (-10.53, 42.36) |
|  | **Placebo** | 96 | 3358.33 (338.09) | -7.93 (-34.21, 18.35) |

**^1^ Number of participants contributing measures at baseline, follow-up or both.**

**^2^ Estimates were derived** using mixed-effects models for repeated measures (MMRM); two-way interactions tested for treatment effect (treatment x time) and APOE ε4 effect (APOE ε4 x time); three-way interactions (treatment x time x APOE ε4) tested whether treatment effects varied by APOE ε4; **; additional covariates were clinic strata, study arm, mean ICV, age and sex.**

***Change in Cognition after the Intervention:***

| **Supplemental Table 4. Treatment Effect on RBANS Scores at 24 Months** | | | |
| --- | --- | --- | --- |
| **Outcome, effect** | **Group** | **Sample size^1^** | **Mean change (95% CI) ^2^** |
| **Total RBANS** |  |  |  |
| **Treatment effect** | **DHA** | 181 | **2.76 (1.15, 4.36)** |
|  | **Placebo** | 183 | 2.67 (1.02, 4.32) |
| **APOE** ε4 **effect** | ε4 **carrier** | 173 | 1.60 (-0.03, 3.24) |
|  | **Non-carrier** | 191 | 3.79 (2.18, 5.40) |
| **Treatment by APOE** ε4 **effect** |  |  |  |
| ε4 **carrier** | **DHA** | 90 | **1.23 (-0.99, 3.44)** |
|  | **Placebo** | 83 | 2.05 (-0.38, 4.48) |
| **Non-carrier** | **DHA** | 91 | 4.45 (2.12, 6.78) |
|  | **Placebo** | 100 | 3.18 (0.94, 5.42) |
| **Immediate memory** |  |  |  |
| **Treatment effect** | **DHA** | 181 | **1.57** (-0.55 , 3.69) |
|  | **Placebo** | 183 | **2.73** (0.55 , 4.90) |
| **APOE4 effect** | ε4 **carrier** | 173 | **-0.66** (-2.78 , 1.45) |
|  | **Non-carrier** | 191 | **4.81** (2.73 , 6.90) |
| **Treatment by APOE** ε4 **effect** |  |  |  |
| ε4 **carrier** | **DHA** | 90 | **-0.67** (-3.54 , 2.20) |
|  | **Placebo** | 83 | **-0.63** (-3.78 , 2.52) |
| **Non-carrier** | **DHA** | 91 | **4.04** (1.02 , 7.06) |
|  | **Placebo** | 100 | **5.53** (2.63 , 8.43) |
| **Delayed memory** |  |  |  |
| **Treatment effect** | **DHA** | 181 | 2.08 (0.29, 3.87) |
|  | **Placebo** | 183 | 3.86 (2.02, 5.70) |
| **APOE** ε4 **effect** | ε4 **carrier** | 173 | 1.94 (0.12, 3.77) |
|  | **Non-carrier** | 191 | 3.92 (2.12, 5.72) |
| **Treatment by APOE** ε4 **effect** |  |  |  |
| ε4 **carrier** | **DHA** | 90 | 1.50 (-0.98, 3.97) |
|  | **Placebo** | 83 | 2.50 (-0.21, 5.22) |
| **Non-carrier** | **DHA** | 91 | 2.73 (0.13, 5.34) |
|  | **Placebo** | 100 | 5.01 (2.51, 7.51) |
| **Attention** |  |  |  |
| **Treatment effect** | **DHA** | 181 | 3.37 (1.28, 5.47) |
|  | **Placebo** | 183 | 0.87 (-1.28, 3.01) |
| **APOE** ε4 **effect** | ε4 **carrier** | 173 | 1.64 (-0.51, 3.79) |
|  | **Non-carrier** | 191 | 2.67 (0.55, 4.78) |
| **Treatment by APOE** ε4 **effect** |  |  |  |
| ε4 **carrier** | **DHA** | 90 | 2.35 ( -0.55, 5.25) |
|  | **Placebo** | 83 | 0.76 (-2.41, 3.94) |
| **Non-carrier** | **DHA** | 91 | 4.50 (1.45, 7.55) |
|  | **Placebo** | 100 | 0.96 (-1.96, 3.89) |
| **Language** |  |  |  |
| **Treatment effect** | **DHA** | 181 | -0.73 (-2.63, 1.18) |
|  | **Placebo** | 183 | -0.10 (-2.06, 1.85) |
| **APOE** ε4 **effect** | ε4 **carrier** | 173 | -1.65 (-3.59, 0.28) |
|  | **Non-carrier** | 191 | 0.77 (-1.14, 2.67) |
| **Treatment by APOE** ε4 **effect** |  |  |  |
| ε4 **carrier** | **DHA** | 90 | -1.97 (-4.61, 0.67) |
|  | **Placebo** | 83 | -1.26 (-4.14, 1.63) |
| **Non-carrier** | **DHA** | 91 | 0.65 (-2.12, 3.42) |
|  | **Placebo** | 100 | 0.88 (-1.78, 3.54) |
| **Visuospatial** |  |  |  |
| **Treatment effect** | **DHA** | 181 | 4.04 (1.53, 6.56) |
|  | **Placebo** | 183 | 2.83 (0.25, 5.40) |
| **APOE** ε4 **effect** | ε4 **carrier** | 173 | 4.82 (2.26, 7.38) |
|  | **Non-carrier** | 191 | 2.12 (-0.39, 4.64) |
| **Treatment by APOE** ε4 **effect** |  |  |  |
| ε4**carrier** | **DHA** | 90 | 3.67 (0.22, 7.13) |
|  | **Placebo** | 83 | 6.14 (2.36, 9.91) |
| **Non-carrier** | **DHA** | 91 | 4.42 (0.81, 8.04) |
|  | **Placebo** | 100 | 0.00 (-3.48, 3.48) |

**^1^ Number of participants contributing measures at baseline, follow-up or both.**

**^2^ Estimates were derived** using mixed-effects models for repeated measures (MMRM); two-way interactions tested for treatment effect (treatment x time) and APOE ε4 effect (APOE ε4 x time); three-way interactions (treatment x time x APOE ε4) tested whether treatment effects varied by APOE ε4; **additional covariates were clinic strata, study arm, education, Hispanic ethnicity, interview language (Spanish/English) and in-person or virtual testing.**

**
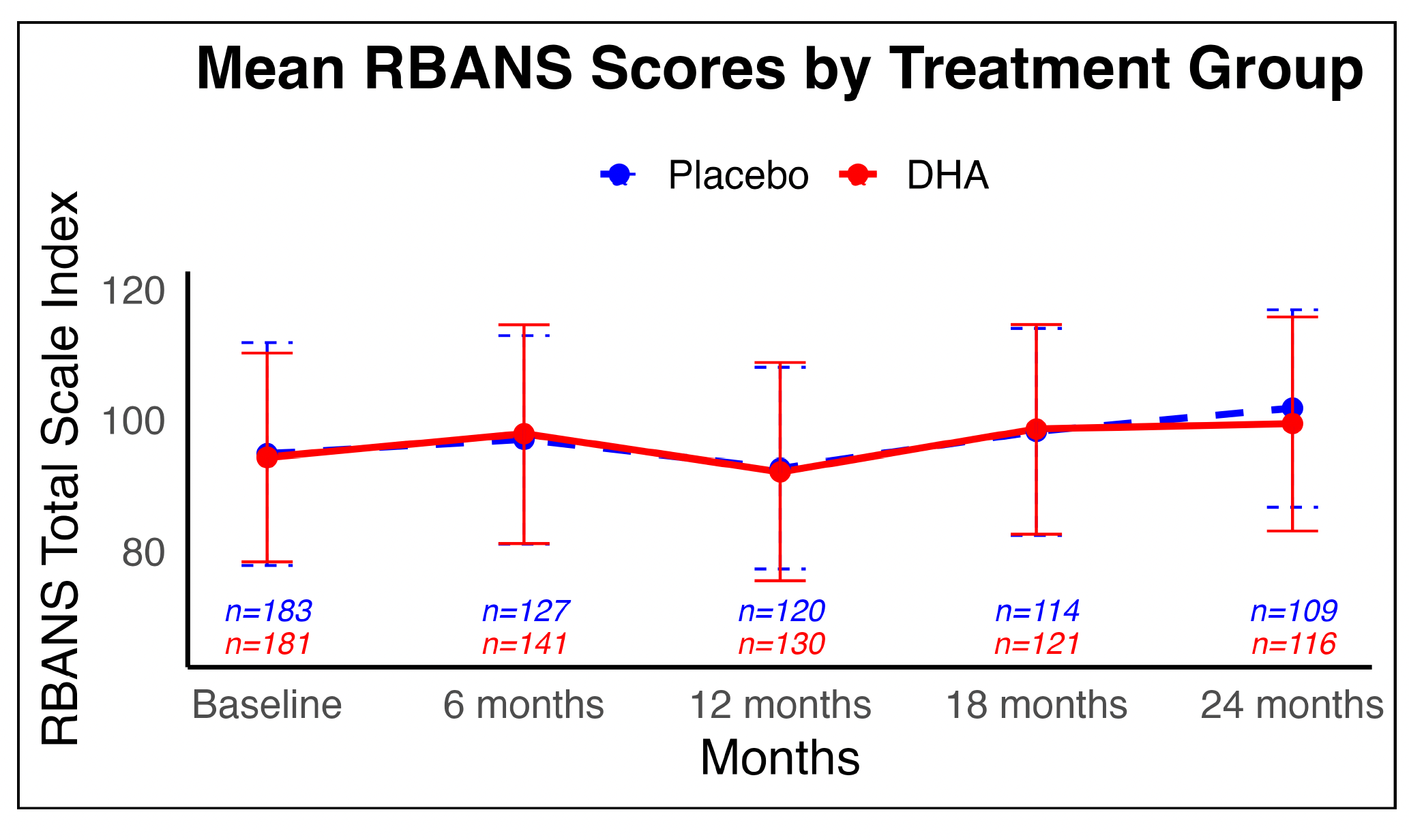
**

**
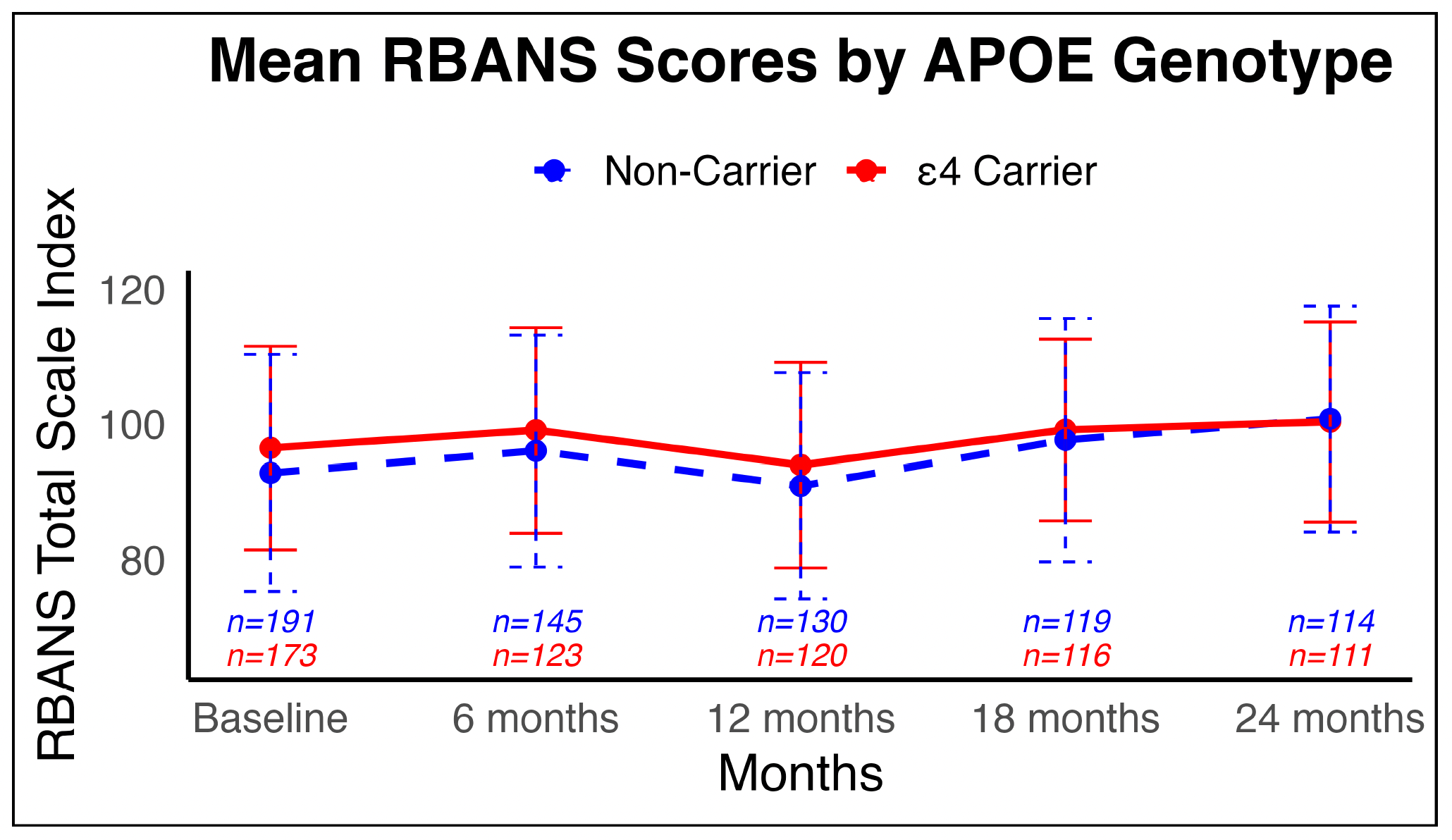
**

**Supplemental Figure 1: Change in RBANS Composite Score Over 24 Months by Treatment and APOE4.** (A) No difference was observed in RBANS mean score change between DHA and placebo groups at 24 months (2·76 [95% CI: **1.15, 4.36**] vs 2·67 [95% CI: 1.02, 4.32];). (B) Effects did not differ by APOE4 carrier status. Mean score change among carriers was 1·23 (95% CI: -0.99, 3.44) for DHA and 2·05 (95% CI: -0.38, 4.48) for placebo ,whereas among non-carriers it was 4·45 (95% CI: 2.12, 6.78) for DHA and 3·18 (95% CI: 0.94, 5.42) for placebo. Error bars represent standard deviations. RBANS=Repeatable Battery for the Assessment of Neuropsychological Status; SD=standard deviation

**Supplementary DHA/AA in Plasma, and RBC section:**

| **Supplemental Table 5. Plasma DHA/AA Change at 6 Months** | | | |
| --- | --- | --- | --- |
| **Group** | **Sample size^1^** | **Mean change (95% CI)^2^** | **Effect size (d)^3^** |
| **Treatment effect** |  |  | 1.65 |
| **DHA** | 131 | 0.49 (0.46, 0.53) |  |
| **Placebo** | 122 | -0.007 (-0.04, 0.03) |  |
| **APOE ε4 effect** |  |  | -0.01 |
| **ε4 carrier** | 119 | 0.24 (0.19, 0.30) |  |
| **Non-carrier** | 134 | 0.25 (0.20, 0.30) |  |
| **Treatment effect by APOE ε4** |  |  | -0.25 |
| **ε4 carrier** |  |  | 1.54 |
| **DHA** | 66 | 0.45 (0.41, 0.50) |  |
| **Placebo** | 53 | -0.01 (-0.06, 0.04) |  |
| **Non-carrier** |  |  | 1.79 |
| **DHA** | 65 | 0.54 (0.49, 0.59) |  |
| **Placebo** | 69 | -0.005 (-0.05, 0.04) |  |

**^1^ Number of participants contributing measures at baseline, follow-up or both.**

^2^ Estimates were derived using mixed-effects models for repeated measures (MMRM); two-way interactions tested for treatment effect (treatment x time) and APOE ε4 effect (APOE ε4 x time); three-way interactions (treatment x time x APOE ε4) tested whether treatment effects varied by APOE ε4; additional covariates were clinic strata and study arm.

^3^ Cohen’s d= group difference in change / standard deviation of change

A

C

B

**Supplemental Figure 2: Intervention Increases Plasma DHA/AA Independent of APOE4 at 6 Months. (A)** DHA increases plasma DHA/AA compared with placebo (mean change 0.49 [95% CI: 0.46, 0.53] vs –0.007 [95% CI: –0.04, 0.03]). **(B)** APOE4 status does not affect the DHA response.

| **Supplemental Table 6a. RBC DHA/AA Change at 24 Months** | | | |
| --- | --- | --- | --- |
| **Group** | **Sample size^1^** | **Mean change (95% CI)^2^** | **Effect size (d)^3^** |
| **Treatment effect** |  |  | 1.48 |
| **DHA** | 92 | 0.46 (0.40, 0.51) |  |
| **Placebo** | 84 | 0.01 (-0.05, 0.07) |  |
| **APOE ε4 effect** |  |  | 0.33 |
| **ε4 carrier** | 83 | 0.30 (0.23, 0.37) |  |
| **Non-carrier** | 93 | 0.20 (0.13, 0.27) |  |
| **Treatment effect by APOE ε4** |  |  | -0.21 |
| **ε4 carrier** |  |  | 1.36 |
| **DHA** | 51 | 0.46 (0.39, 0.54) |  |
| **Placebo** | 32 | 0.05 (-0.03, 0.14) |  |
| **Non-carrier** |  |  | 1.57 |
| **DHA** | 41 | 0.45 (0.37, 0.53) |  |
| **Placebo** | 52 | -0.03 (-0.10, 0.05) |  |

**^1^ Number of participants contributing measures at baseline, follow-up or both.**

^2^ Estimates were derived using mixed-effects models for repeated measures (MMRM); two-way interactions tested for treatment effect (treatment x time) and APOE ε4 effect (APOE ε4 x time); three-way interactions (treatment x time x APOE ε4) tested whether treatment effects varied by APOE ε4; additional covariates were clinic strata and study arm.

^3^ Cohen’s d= group difference in change / standard deviation of change


**Supplemental Figure 3a : Intervention Increases RBC DHA/AA Independent of APOE4. (A)** DHA increased RBC DHA/AA compared with placebo (0.46[0.40, 0.51] 0.01 [–0.05, 0.07]). **(B)** Mean changes were similar in APOE4 carriers (0.46 [0.39, 0.54] vs 0.05[–0.03, 0.14];) and non-carriers (0.45[0.37, 0.53] vs –0.03 [–0.10, 0.05]).

B

A

| **Supplemental Table 6b. RBC Omega-3 Index (%) Change at 24 Months** | | | |
| --- | --- | --- | --- |
| **Group** | **Sample size^1^** | **Mean change (95% CI)^2^** | **Effect size (d)^3^** |
| **Treatment effect** |  |  | 1.68 |
| **DHA** | 92 | 6.18 (5.46, 6.90) |  |
| **Placebo** | 84 | 0.21 (-0.54, 0.96) |  |
| **APOE ε4 effect** |  |  | 0.42 |
| **ε4 carrier** | 83 | 4.14 (3.16, 5.12) |  |
| **Non-carrier** | 93 | 2.66 (1.70, 3.62) |  |
| **Treatment effect by APOE ε4** |  |  | -0.32 |
| **ε4 carrier** |  |  | 1.50 |
| **DHA** | 51 | 6.24 (5.26, 7.21) |  |
| **Placebo** | 32 | 0.90 (-0.24, 2.03) |  |
| **Non-carrier** |  |  | 1.82 |
| **DHA** | 41 | 6.13 (5.08, 7.19) |  |
| **Placebo** | 52 | -0.34 (-1.34, 0.67) |  |

**^1^ Number of participants contributing measures at baseline, follow-up or both.**

^2^ Estimates were derived using mixed-effects models for repeated measures (MMRM); two-way interactions tested for treatment effect (treatment x time) and APOE ε4 effect (APOE ε4 x time); three-way interactions (treatment x time x APOE ε4) tested whether treatment effects varied by APOE ε4; additional covariates were clinic strata and study arm.

^3^ Cohen’s d= group difference in change / standard deviation of change

B


A

**Supplemental Figure 3b : Change in Red Blood Cell (RBC) Omega-3 Index (%) by Treatment Group and APOE4.** **(A)** DHA supplementation increased the Omega-3 Index compared with placebo (mean change 6.18[95% CI: 5.46, 6.90] vs 0.21[95% CI: –0.54, 0.96];).**(B)** APOE4 carrier status did not alter the treatment effect.

B
