# Supplementary material for: CNS target engagement of high-dose DHA supplementation in older adults at risk for dementia: a randomised, double-blind, placebo-controlled trial
Source: eBioMedicine. 2026 Jun 18;129:106316. doi: 10.1016/j.ebiom.2026.106316 (PMC13347776; doi:10.1016/j.ebiom.2026.106316)
Supplement: Protocol [file mmc2.docx]

**PreventE4 Trial**

**Principal Investigator:**

Hussein Yassine

University of Southern California

2250 Alcazar st, rm 210

Los Angeles, CA, 90033

Cell: 216-526-6316

**Co-Investigators:**

Lon Schneider

Howard Hodis

Wendy Mack

Meredith Braskie

**CONFIDENTIAL**

**Amendment Summary**

This protocol combines the two arms of the PreventE4 trial, the Brain DHA Delivery Trial (primary endpoint is the CSF DHA/AA change at 6 months, funded by NIH R01 AG054434, 2018-2024, IRB, IRB# HS-18-00291, n=184) and cognition/imaging arm at 2 years (funded by ADDF GC-201711-2014197 2019-2024, IRB# HS-18-00984, n=368) with identical procedures for both arms with the exception of the lumbar puncture (LP) at 6 months in the NIH funded study. Since the start of the trial, the major amendments were 1) increasing in sample size from 320 to 368 due to the COVID pandemic greater than expected dropout rate, 2) conducting some of the neuropsychology testing online or by phone during the COVID pandemic 3) addition of stool sample collection to study the microbiome and 4) adding a post study extension follow-up examine the effect of the intervention on cognition after the study completion. This protocol reports the trial outcomes, statistical analysis, sample size adjustments and protocol changes.

**TABLE OF CONTENTS**

TITLE PAGE 1

AMENDMENT 2

TABLE OF CONTENTS 3

ABBREVIATIONS 5

SYNOPSIS 7

1. ABSTRACT 9

1.1 Specific Aims and Objectives

2. BACKGROUND and SIGNIFICANCE 9

2.1 Alzheimer’s: The Problem and its Importance

2.2 APOE4: Detrimental Effects on Alzheimer’s disease

2.3 DHA: Essential Fatty acid for Neuronal Functions

2.4 Rationale for the Intervention

2.5 Supporting data

3. STUDY DESIGN 14

3.1 Inclusion/Exclusion Criteria

3.2 Endpoints

3.3.1 Brain Imaging by MRI

3.3.2 Neuropsychological Assessments

3.3 Treatments

3.4.1 Placebo vs 2 grams of DHA

3.4.2 Vitamin B complex

3.4.3 FDA Compliance

4. RECRUITMENT and RANDOMIZATION 17

4.1 Sources of Participants

4.2 Recruitment and Screening

4.3 Informed Consent

4.4 Baseline Testing and Follow-Up Visits

4.5 Randomization

4.6 Masking

5. PARTICIPANT MANAGEMENT 20

5.1 Overview

5.2 Drug Distribution

5.3 Adherence Measures

5.4 Retention

6. DATA COLLECTION 21

6.1 Pre-screening and Screening Eligibility Measurements

6.2 Main Study Procedures

6.2.1 Dietary and Physical Activity Advice During Pre-screening

6.2.2 Fasting Blood Sample

6.2.3 ApoE Genotyping

6.2.3 Physical Measures

6.2.4 Questionnaires

6.2.5 Brain Imaging

6.2.6 Safety Measures

6.2.7 GUID

7. SAFETY/HUMAN SUBJECTS PROTECTION 25

7.1 Data and Safety Monitoring Board (DSMB)

7.2 Safety Review Plan and Monitoring

7.3 Confidentiality

7.4 Expected Side Effects

7.5 Risk Management

7.5.1 DHA

7.5.2 Adipose tissue

7.6 HIPAA – Protection of Patient Information

8. ADVERSE EVENT REPORTING 28

8.1 Definition of Serious Adverse Events

8.2 Non-serious Adverse Events

8.3 Reporting Adverse Events

8.4 Tracking Adverse Events

8.5 Emergency Unmasking

9. STATISTICAL CONSIDERATIONS 29

9.1 Outcomes and Analyses

9.2 Sample Size/Power Calculations

9.3 Data Analysis

10. DATA PROCESSING and MANAGEMENT 31

10.1 Data Management System

10.2 Data Transfer

10.2 Quality Control

10.4 Back-up, Data Security and Confidentiality

10.5 Tracking Study Progress

10.6 Archiving and Study Close-out Including Repository

11. STUDY ADMINISTRATION 33

11.3 Training and Certification

11.6 Conflict of Interest Policy

11.7 Publications and Presentations Policy

11.9 Protocol Amendments

12. STUDY TIMELINE 34

13. COMPENSATION 34

15. LITERATURE CITED 35

**ABBREVIATIONS**

AA Arachidonic acid

APOE/APOE4 Apolipoprotein (APOE) epsilon 4 (APOE4)

Aβ Beta Amyloid

BUN Blood Urea Nitrogen

CDR-SOB Clinical Dementia Rating – Sum of Boxes

CSF Cerebrospinal Fluid

DHA Docosahexaenoic Acid

DHASCO Docosahexaenoic Acid-Rich Single-Cell Oil

DHHS Department of Health and Human Services

DNA Deoxyribonucleic Acid

DSMB Data Safety Monitoring Board

DSM-IV Diagnostic and Statistical Manual of Mental Disorders, Fourth Edition

ECG Electrocardiogram

EDTA Ethylene Diamine Tetra Acetic Acid

ELISA Enzyme-Linked Immunosorbent Assay

EPA Eicosapentaenoic Acid

FDA Food and Drug Administration

FFQ Food Frequency Questionnaire

HCT Hematocrit

HDL High-density Lipoproteins

HGB Hemoglobin

HIPAA Health Insurance Portability and Accountability Act

ICF/ICD Informed Consent Form/Document

IRB Institutional Review Board

ITT Intent to Treat

MCV Mean Corpuscular Volume

MMSE Mini Mental State Examination

MR/MRI Magnetic Resonance / Magnetic Resonance Imaging

NIA National Institute on Aging, under the NIH

NIH National Institutes of Health

PC Phosphatidylethanolamine

PE Phosphatidylethanolamine

PHI Protected Health Information

PI Principal Investigator

PLA_2_ phospholipase A_2_

PUFA Polyunsaturated Fatty Acid

QA Quality Assurance

QC Quality Control

**SYNOPSIS**

| Title | **PreventE4** |
| --- | --- |
| Protocol Committee | Hussein Yassine, MD—Principal Investigator  Lon Schneider, MD  Howard Hodis, MD  Meredith Braskie, PhD  Wendy Mack, PhD |
| Study Sponsor | NIA, ADDF |
| Study Phase | Phase IIa |
| Indication | Non-demented individuals |
| Primary objective | To test the interaction of the DHA treatment effect on the change in CSF DHA/AA by APOE4 genotype at 6 months in the LP subset (n=184). |
| Secondary Objectives | Test the effect of DHA treatment on the change of resting state functional MRI, DTI and volumetric MRI in all 368 participants at 2 years. |
| Exploratory | Test the effect of DHA treatment on the change of cognition (RBANS), plasma eicosanoids, oxylipins, abeta 42/40, ptau 181 at 2 years  Stool sample change at 6 months and 24 months for microbiome analysis |
| Study Design | Double-blind, placebo-controlled randomized clinical trial in 368 non-demented individuals of 2 grams per day of DHA vs. Placebo over 24 months stratified by APOE4 carrier status |
| Study Endpoints, Subsets and Covariates | Primary endpoint is the change in CSF DHA/AA at 6 months, secondary outcome is the change in brain imaging outcomes after 24 months in all study participants, and exploratory outcomes include the change in cognitive outcomes and plasma biomarkers after 24 months in all study participants, and stool biomarkers after 24 months in small subset of study participants |
| Sample Size | 368 (184 DHA and 184 Placebo, 1:1 randomization), stratified by APOE4 carrier state |
| Summary of Key Eligibility Criteria | - Age: 55-80  - At least one CVD dementia risk factor  - Age adjusted Mini-Mental State Examination (MMSE) ≥ 25.  - Logical Memory II delayed recall score ≥ 6 and ≤ 18.  -Not taking ω-3 supplements for past 3 months  - DHA consumption of less than 200 mg/day |
| Drug, Dosage and Formulation | 2 grams of DHA will be administered in a divided dose,  twice daily, to be given with food to minimize GI effects. |
| Placebo | A matching placebo (corn/soy oil) will be used. |
| Route of Administration | Oral |
| Procedures | Physical exam, MRI, RBANS, FFQ, DHA and exercise questionnaire |
| Statistical considerations | Sample size was estimated to test an interaction hypothesis between DHA intervention and APOE4 genotype (i.e., that the DHA intervention effect on CSF DHA/AA will differ by APOE4 genotype) at 80% power. The interaction effect size (standard deviation of interaction mean effects, divided by pooled between subject SD) of 0.25 relates to a 50% difference in the DHA effect in APOE4 positive compared to APOE4 negative individuals. To obtain 80% power considering the 30% dropout, the sample size was 184 (46 per cell) for the LP arm of the trial. The secondary and exploratory trial outcomes will be assessed in the combined sample (LP and no-LP arms) of 368 participants to detect an effect size of 0.5 SD for the main effect of DHA compared to placebo with a 30% dropout.  The primary outcome of CSF DHA/AA will be evaluated in the LP arm using a general linear model that includes main effects of randomized treatment, the APOE4 carrier and clinical site stratification factors, and the interaction between APOE4 stratum and treatment group.  The difference in cognitive and MRI changes at 24 months will be analyzed, α=0.05 with no adjustment for multiple comparisons. The results will be reported as point estimates (of main effect treatment group differences, overall and by APOE4 status) with corresponding confidence intervals.  All outcome measures (changes in CSF DHA/AA, imaging, cognition and biomarkers) will be analyzed using the same general linear model. If we find a significant group difference (p<0.05) in the primary or secondary outcomes (ε4 carriers vs. non-carriers and supplemented vs. placebo), we will conduct analyses to identify potential mediation of the treatment effect. |

**1 ABSTRACT**

The APOE ε4 allele is the strongest genetic risk factor for developing Alzheimer's disease (AD). DHA is an essential omega-3 fatty acid critical to neuronal functions and is not formed in sufficient amounts *de novo*. DHA is highly enriched in cortical grey matter and is more concentrated at synapses where it plays a role in synaptic plasticity. DHA is depleted in AD brains. Randomized clinical trials have yielded mixed results on the effect of DHA on cognitive outcomes.

We hypothesize that APOE ε4 carriers have reduced delivery of DHA to the brain that can be reversed upon high dose DHA supplementation. To address this hypothesis, we propose a double-blind placebo-controlled clinical trial of high dose (2 grams/day) of DHA over 24 months in 368 cognitively healthy participants stratified by APOE status (ε4 vs. non ε4 carriers). The primary outcome is the effect of APOE genotype on CSF DHA/AA changes at 6 months in 184 participants. The secondary outcomes are the changes in imaging biomarkers in all participants. The exploratory outcomes include changes in cognition and plasma biomarkers at 24 months in all participants and stool biomarkers at 6 and 24 months in small subset of study participants.

The results of these studies will provide novel information that can be used clinically to design personalized approaches for the prevention of AD in high-risk individuals. A study extension is added to examine cognitive change yearly for 3 years after the study completion.

- 1. **Specific Aims and Objectives**

The objective of the proposed project is to test if carriers of the APOE4 allele of the APOE gene improve their cognition and brain connectivity after DHA supplementation, compared to persons who do not carry the APOE4 allele. The implications of this study are significant to the field of AD. If our hypothesis is confirmed, our long-term goal is to determine whether higher levels of DHA intake for APOE4 carriers, who are at elevated risk for AD, could prevent cognitive decline.

Specific Aims: We hypothesize that APOE4 carriers at risk of cognitive decline will have a defect in the delivery of DHA to the central nervous system after supplementation. To address this hypothesis, our specific aim is to recruit 184 carriers and 184 non-carriers of APOE4 allele for DHA supplementation over 24 months. Participants will be given placebo or 2 grams of DHA/day (n=92 per group).

Our outcomes are 1. Changes in CSF DHA/AA at 6 months, n=184, 2. Changes in brain imaging at 24 months in all study participants, n=368. 3. Changes in cognition and plasma biomarkers at 24 months in all study participants, n=368 and stool biomarkers at 24 months in small subset of study participants, n=30.

A study extension is added to study the effect of the intervention on cognition for 3 years after the completion of the study.

1. **BACKGROUND and SIGNIFIGANCE**

**2.1 Alzheimer’s Disease: The Problem and its Importance**

Alzheimer's disease (AD) is one of the most persistent and devastating dementing disorders of old age, because it eventually leads to a complete loss of memory and of the ability to function independently. It is estimated that over five million people in the United States have AD in its various stages at an estimated cost to society of over $200 billion per year, and it is projected that 11 to 16 million people and their families could be affected by AD by the middle of this century. To date, no effective therapies are developed to prevent, slow or stop the disease. In order to develop effective therapies, mechanistic studies of AD are urgently needed.

**2.2 APOE4: Detrimental Effects on Alzheimer’s Disease**

Among 19 susceptibility genes identified for AD, apolipoprotein E4 (APOE4) is by far the strongest genetic risk factor for Alzheimer disease [[1](#_heading=h.1hmsyys)]. Humans can express 3 alternative isoforms of apo E – E2, E3 and E4 – the most common of which is apo E3. The ε4 allele frequency in the general population is 15%, but in up to 50% of patients with AD. Individuals with one ε4 allele are 3 to 4 times as likely to develop AD as those without any ε4 allele, and people with two ε4 alleles have a 12-fold higher risk of developing AD (reviewed in [[2](#_heading=h.41mghml)]). Moreover, the age of onset of AD symptoms is earlier in carriers of the ε4 allele than in those with the ε3 allele. On the other hand, carriers of the ε2 allele have decreased risk of developing AD [[3](#_heading=h.2grqrue)]. ApoE is a major lipoprotein in the CNS (brain and CSF) and exchanges cholesterol and lipids between astrocytes and neurons [[4](#_heading=h.vx1227), [5](#_heading=h.3fwokq0)]. This function is isoform specific; APOE4 particles are less capable of this exchange compared to E3 or E2 [[4](#_heading=h.vx1227)].

**2.3 Docosahexaenoic acid (DHA): Essential Fatty Acid for Neuronal Functions**

DHA is considered an essential PUFA as *de novo* production at sufficient quantities is not possible in humans. The brain requires essential fatty acids (such as DHA) for maintenance of neuronal membranes, clearance of brain waste such as abeta amyloid (Aβ) protein, and modulation of inflammation [[6](#_heading=h.1v1yuxt)].

**2.4 APOE4 and DHA metabolism**

Our studies suggest that APOE4 carriers have abnormalities in brain DHA metabolism that precede the onset of Alzheimer’s disease (AD) dementia [[7](#_heading=h.4f1mdlm)]. Using DHA PET imaging, we demonstrated in young and middle-aged APOE4 carriers an increased avidity for [^11^C] DHA in AD susceptible brain regions compared with non-carriers [[8](#_heading=h.2u6wntf)]. This suggests a brain DHA deficit with APOE4 that appears before the onset of dementia. Decreased brain DHA levels are observed in 1-year old human ApoE4 (APOE4) targeted replacement (TR) mice compared to non-E4 TR mice [[9](#_heading=h.19c6y18)].

There are multiple mechanisms for this brain DHA deficit with APOE4, including defects in brain APOE4 lipid metabolism, transport across the blood-brain barrier, or abnormal liver and adipose tissue fatty acid metabolism-reviewed in [[7](#_heading=h.4f1mdlm), [10](#_heading=h.3tbugp1)]. One of the largest DHA stores in the body is the adipose tissue [[11](#_heading=h.28h4qwu)]. APOE4 TR mice fed an ω-3 deficient diet have 45% less adipose DHA levels compared with APOE3 TR mice [[12](#_heading=h.nmf14n)]. This abnormality in DHA metabolism makes APOE4 carriers more susceptible to dietary DHA deficiency than the general population [[10](#_heading=h.3tbugp1)].

Importantly, two independent groups demonstrated that early and long term high dose DHA supplementation in APOE4 animal models prevented cognitive deficits [[13](#_heading=h.37m2jsg), [14](#_heading=h.1mrcu09)]. In these studies, enriching the diet with a relatively high dose of DHA (the equivalence of 3 grams per day in humans) for several weeks was associated with preserved cognitive functions in middle-aged APOE4 mouse models. The rodent studies highlight the importance of early and high dose DHA supplementation in APOE4. Subgroup analyses of three randomized clinical trials are consistent with these rodent studies and suggest that APOE4 carriers may benefit from ω-3 supplementation when started prior to the onset of cognitive impairment (Table 1) and reviewed in [[7](#_heading=h.4f1mdlm)]. This question has not yet been directly tested in clinical trials and is important given that approximately 20% of the American population carries the APOE4 allele. In contrast, DHA supplementation after the onset of dementia or cognitive impairment does not appear to be beneficial. For example, in the ADCS-sponsored DHA trial, DHA supplementation in mild AD was not associated with cognitive benefit. These studies are summarized in table 1.

| **Table 1. Subgroup analysis for the effect of APOE genotype in ω-3 randomized clinical trials** | | | | |
| --- | --- | --- | --- | --- |
| **Study** | **Average Age** | **Setting** | **ω-3 trial design** | **Beneficial signal in selected outcomes** |
| Quinn et al [[15](#_heading=h.46r0co2)] | 76 | AD | 2000 mg/d DHA vs. placebo over 18 m improved ADAS cog and MMSE | APOE4 non-carriers |
| Stonehouse et al [[16](#_heading=h.2lwamvv)] | 33 | CH | 1160 mg/d DHA vs placebo over 6 m improved memory retention time | APOE4 carriers |
| Andrieu et al [[17](#_heading=h.111kx3o)] | 75 | CH | 800 mg/d DHA with multidomain intervention vs. placebo over 36 m improved MMSE orientation score | APOE4 carriers |
| Van de Rest [[18](#_heading=h.3l18frh)] | 70 | MCI | 1800 mg/d EPA–DHA vs. placebo over 6 m improved attention domain | APOE4 carriers |
| Yurko-Mauro [[19](#_heading=h.206ipza)] (E4 genotyping was not done) | 70 | CH | 900 mg/d DHA vs. placebo over 6 m improved learning and verbal memory | Positive effect on memory. Largest effect in persons with family history of dementia |
| AD: Alzheimer’s disease, CH: Cognitively Healthy, MCI: Mild Cognitive Impairment, m:month, d:day, mg: milligram | | | | |

APOE4, DHA dose and brain penetrance: The association of ω-3 consumption and APOE4 on cognitive outcomes in observational cohorts has been inconsistent. For example, in some observational studies, benefit of increased seafood or ω-3 consumption on cognition or AD incidence was restricted to APOE4 non-carriers [[20-23](#_heading=h.4k668n3)]. Our studies suggest that APOE4 carriers have reduced brain DHA delivery [[9](#_heading=h.19c6y18), [24](#_heading=h.2dlolyb)]. We hypothesize that lower dose ω-3 supplementation (< 1 gram per day) would not translate into efficient brain DHA delivery in APOE4 carriers explaining the lack of benefit of ω-3 supplementation in these studies. Using DHA does of > 1 gram per day has been associated with greater drug-placebo differences on cognitive outcomes in some randomized clinical trials [[25](#_heading=h.sqyw64)].

Defining the target population: One challenge of testing interventions to prevent AD in non-cognitively impaired populations is the heterogeneity of the sample population with the variable rate of cognitive deterioration. Although APOE4 is a major genetic risk factor for AD, the risk of cognitive deterioration in APOE4 carriers varies, and increases with increased cardiovascular disease risk (CVD) factors, decreased education, increased age, and limited seafood consumption. This is evident in the Stockholm longitudinal cohort [[31](#_heading=h.3q5sasy)], the Finnish cohorts [[32](#_heading=h.25b2l0r)], the Rush Memory and Aging Project [[33](#_heading=h.kgcv8k)], and many other studies [[34](#_heading=h.34g0dwd)]. We believe that targeting cognitively normal APOE4 carriers with CVD dementia risk factors but before the onset of significant cognitive impairment and neurodegeneration is critical for the success of AD prevention strategies [[7](#_heading=h.4f1mdlm), [35](#_heading=h.1jlao46), [36](#_heading=h.43ky6rz)].

**2.5 Supporting data**

**Figure 1:** The effect of DHA treatment vs. placebo on CSF DHA levels by APOE genotype in persons with dementia. The increases in DHA levels in the CSF were less pronounced in carriers of the ɛ4 allele. There was a suggestion for an interaction effect between APOE genotype and treatment arm on CSF DHA levels (p=0.07).


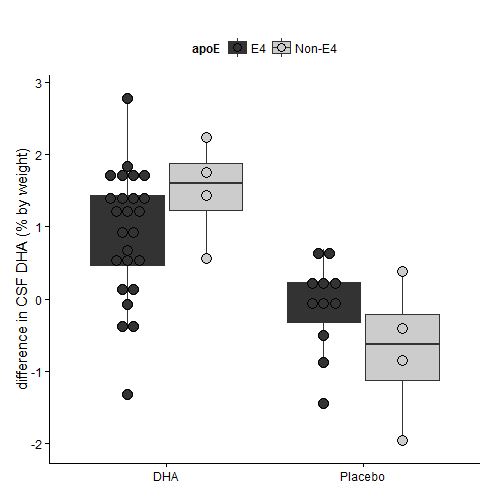


The data gathered in this application suggest that APOE4 carriers might have reduced delivery of DHA to the brain. These data support DHA supplementation for APOE4 carriers and for persons at risk of AD.

APOE4 status and cerebrospinal (CSF) DHA: Our research group has shown that participants with MCI and AD have decreased DHA levels in the CSF [[37](#_heading=h.2iq8gzs)]. In a randomized clinical trial that examined the effect of 2 grams per day of DHA vs. placebo on cognitive outcomes in AD [[15](#_heading=h.46r0co2)], non-carriers of APOE4 had significant improvements in the Alzheimer’s Disease Assessment Scale (ADAS-cog) score after DHA intake (n=91) compared to placebo (n=64). No difference in ADAS-cog scores was observed in APOE4 carriers allocated to DHA (n=137) compared to placebo (n=95). 28 participants (non-APOE4 carriers, n=4 and APOE4 carriers, n=24) had measures of DHA in the CSF at baseline and at 18 months after allocation to 2 grams/day of DHA treatment. We found less increase in CSF DHA levels after 18 months of DHA treatment in APOE4 carriers compared to non-carriers (**Figure 1**)[[24](#_heading=h.2dlolyb)].

Amyloid pathology and DHA brain delivery: One hallmark of APOE4 is increased brain amyloid deposition that precedes cognitive impairment and is indirectly assessed by lower CSF Aβ42 levels [[38](#_heading=h.xvir7l)]. To determine whether amyloid pathology influences the penetration of DHA in the brain, Calon et al measured cerebral uptake of [^14^C]-DHA in 3xTg-AD mice (prone to brain amyloid deposition). The investigators found a 25% (p < 0.001) decrease of brain transport coefficients of [^14^C]-DHA in this model of AD, compared to non-transgenic littermates [[39](#_heading=h.3hv69ve)]. We investigated whether brain amyloid pathology affected DHA transport to CSF in the ADCS DHA study [[24](#_heading=h.2dlolyb)]. We found that lower CSF Aβ42 was associated with less increase is CSF DHA levels after supplementation (**Figure 2**). These findings support understanding the mechanisms of how amyloid pathology alters DHA brain delivery. We predict that cognitively healthy older adults with lower CSF Aβ42 will have decreased DHA brain delivery supporting the rationale for DHA supplementation with APOE4.


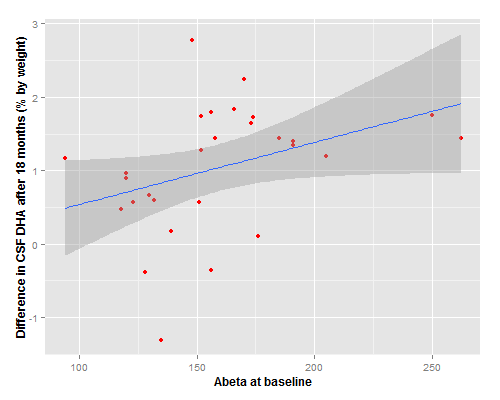


**Figure 2:** Baseline CSF Aβ42 levels were significantly associated with the CSF DHA change after supplementation (r=0.37, p=0.05) from Yassine et al, 2016).

Effect of DHA and APOE4 genotype on brain structural and functional connectivity: In the Aging Brain Study, we found that lower serum DHA levels correlated with lower hippocampal and entorhinal volumes independent of APOE genotype [[40](#_heading=h.1x0gk37)]. Greater serum DHA levels were associated with preserved hippocampal and entorhinal volumes in both carriers and non-carriers of APOE4. However, APOE4 carriers have distinct changes in brain volumes, structural and functional connectivity compared to non-carriers before any manifestations of cognitive changes. For example, the entorhinal cortex (ERC) is one of the earliest sites of neurofibrillary tangle deposition in non-demented older adults [[41](#_heading=h.4h042r0)] and is thinner in healthy older adults who carry the APOE4 allele versus non-carriers [[42](#_heading=h.2w5ecyt)]. In previous ω-3 studies [[23](#_heading=h.3ygebqi), [43](#_heading=h.1baon6m)], hippocampal volume was affected. In a retrospective study of brain volume and ω-3 use in ~800 participants (healthy, MCI and AD) over 4 years [[23](#_heading=h.3ygebqi)], ω-3 use was associated with increases in hippocampal volumes. Other APOE4-related differences have been identified in healthy adults in subicular cortex thickness [[42](#_heading=h.2w5ecyt)] resting state fMRI synchronicity of default mode network (DMN) signal [[44](#_heading=h.3vac5uf), [45](#_heading=h.2afmg28)] and white matter integrity [[46](#_heading=h.pkwqa1)]. Since DHA is enriched in oligodendrocytes as well as neurons, it might play a role in myelin structure [[47](#_heading=h.39kk8xu)] and in the brain’s functional connectivity. Indeed, the study by Witte et al., found that 26 weeks of taking 2 grams per day of ω-3s resulted in more intact white matter in the inferior and superior longitudinal fasciculi (ILF; SLF), inferior fronto-occipital fasciculus (IFO), and corpus callosum [[43](#_heading=h.1baon6m)]. These regions, in addition to cingulum, have previously shown white matter integrity differences in healthy adult APOE4 carriers versus non-carriers using diffusion MRI (dMRI) [[46](#_heading=h.pkwqa1), [48-50](#_heading=h.1opuj5n)]. Finally, mirroring results in AD patients versus controls [[51](#_heading=h.1302m92)], in healthy adults, APOE4 has been associated with lower synchronicity of rs-fMRI signal between the hippocampus and the DMN, a network of regions that are more active at rest than during an effortful task [[44](#_heading=h.3vac5uf), [52](#_heading=h.3mzq4wv)]. Combined, these studies support examining the effect of DHA supplementation on brain volumes, structural and functional connectivity by APOE genotype before the onset of dementia. We propose to investigate whether some of these differences might relate to deficits in DHA delivery to the brain or brain DHA metabolism.

1. **STUDY DESIGN**


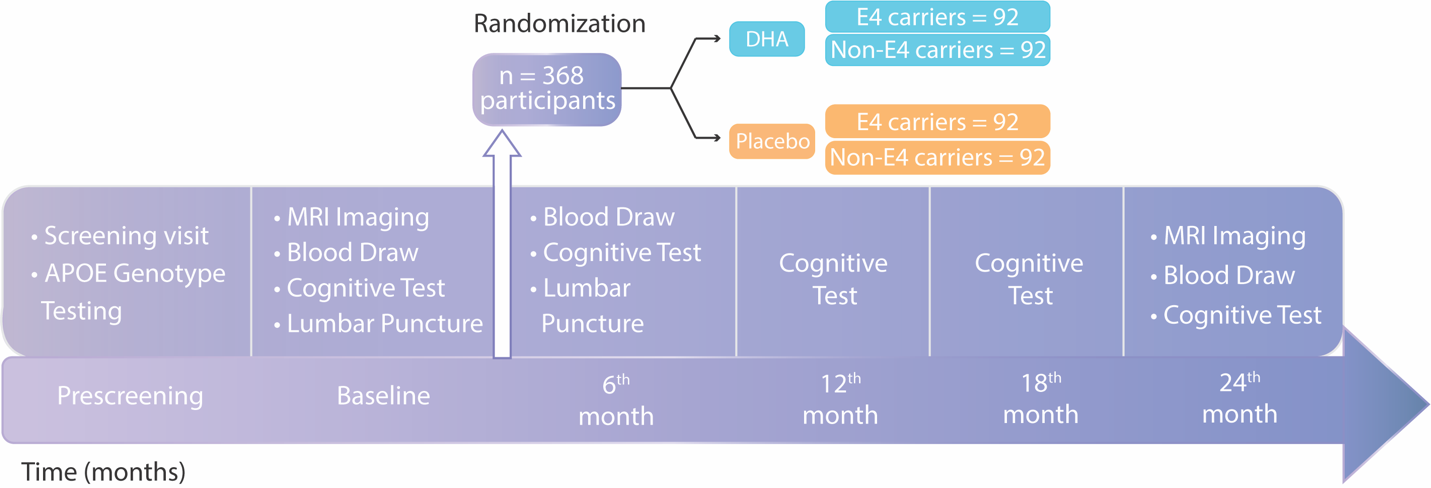


**3.1 Overall design**

For this study, we propose to recruit a total 368 cognitively non-impaired adults stratified 1:1 by APOE4 genotype (Figure 3). The definition of cognitively non-impaired is based on ADNI criteria with the following inclusion and exclusion criteria.

| **Table 2: CVD Dementia risk factors** | |
| --- | --- |
| **Risk factors** | **Cut-off** |
| BMI | >30 kg/m^2^ |
| BP | >140/90 mmHg |
| Total cholesterol | >250 mg/dL |
| Physical inactivity | Less than 30 min three times per week of physical activity |
| Education years | <12 years |

Inclusion Criteria:

- Age: ≥ 55 and ≤ 80

- At least one CVD dementia risk factor (Table 2)

- Age adjusted Mini-Mental State Examination (MMSE) ≥ 25 (allowing for education adjustment)

- Logical Memory II delayed recall score ≥ 6 and ≤ 18 (allowing for education adjustment).

Exclusion Criteria:

- No clinical evidence of dementia as assessed by DSM dementia criteria assessed by the investigator

- FAQ questionnaire score ≥ 9 and indicating 3 or more areas of impaired functioning, or dementia as assessed by the investigator

- Use of ω-3 preparations over the last 3 months

- > 200 mg/day of DHA consumption using a validated questionnaire

- Historical use of donepezil, rivastigmine, galantamine and/or memantine

- Alcohol or drug abuse

- A concomitant serious disease such as active cancer treatment or HIV.

- Major mood disorders including depression and bipolar disorders (DSM-IV)

- Participation in any other clinical trial in the last 30 days

**3.2 Outcomes**

***Primary outcomes:*** The primary trial outcome measure is the 6-month change in cerebrospinal fluid DHA/AA in 184 subjects consenting to lumbar puncture (LP). Baseline and 6-month cerebrospinal fluid DHA/AA will be assessed by mass spectrometry.

***Secondary outcomes:*** The secondary outcome measures include the 2-year change in functional and structural connectivity using resting state functional MRI and brain volumetrics in all 368 participants*.*

***Exploratory outcomes:*** The exploratory outcomes include changes in cognition and plasma and stool biomarkers. Selection of the exploratory cognitive outcome for this trial requires identifying the domains most relevant to both the metabolic profile of the selected population and sensitive to enhancement by the intervention of DHA supplementation. AD clinical trials have attempted to identify several composite scores (such as the preclinical Alzheimer cognitive composite PACC [[53](#_heading=h.2250f4o)], Neuropsychological Test Battery (NTB) [[54](#_heading=h.haapch)], Repeatable Battery for the Assessment of Neuropsychological Status (RBANS) composite scores [[55](#_heading=h.319y80a)]). These composites were developed in one cohort and shown to have sensitivity in detecting early cognitive changes in preclinical or prodromal AD. However, change over time beyond test populations has been limited contributing to loss of study power [[35](#_heading=h.1jlao46), [36](#_heading=h.43ky6rz)].

To address this challenge in the current proposal, we focused on what is known about the cognitive profile in those with APOE4 positivity [[56](#_heading=h.1gf8i83)], and early aging changes as well as what is improved in the best case scenario of DHA enhancement [[19](#_heading=h.206ipza)]. These tests may not fully overlap with tests selected for AD trials as they are selected for purpose rather than similarity to other disease. For example, among non-demented APOE4 carriers and over the age 60, verbal memory shows the greatest likelihood of decline compared to visuospatial awareness and general mental status [[56](#_heading=h.1gf8i83)]. In addition, there is modest evidence of an allele dose effect [[56](#_heading=h.1gf8i83)]. In terms of the effect of DHA in non-demented adult and aging populations, the domain most likely to show benefit is verbal memory [[19](#_heading=h.206ipza)]. A second major issue is ensuring that the cognitive assessment is repeatable and not overly sensitive to practice effect, particularly in those who are not cognitively impaired. To this end, the RBANS immediate memory and delayed memory scale index scores have minimal ceiling effect, designed for clinical trials, and captures domains that previously demonstrated response to omega-3 supplementation [[16](#_heading=h.2lwamvv), [18](#_heading=h.3l18frh), [19](#_heading=h.206ipza), [57](#_heading=h.40ew0vw)]. The RBANS is available in validated computerized format and will be integrated within a single battery.

We propose to assess the domain specific composite for memory (both RBANS immediate and delayed memory scale) as the primary outcome.

Other exploratory outcomes include (1) attention/executive domain (assessed through reaction time/information processing speed/conceptual shifting/selective attention assessed in the Trails A and B tests, and Coding (RBANS), (2) working memory (Digit Span, RBANS) (3) visuospatial analysis-RBANS, language-RBANS, and (4) MMSE orientation score (selected based on based on MAPT [[17](#_heading=h.111kx3o)], table 1). We will also obtain Clinical Dementia Rating scores (CDRs) given a potential signal in LipiDiDiet [[54](#_heading=h.haapch)], although we do not expect major changes in CDR sum of boxes in these cognitively non-impaired individuals over two years, plasma biomarkers of inflammation (eicosanoids and oxylipins), and amyloidosis (plasma abeta 42/40, ptau 181) in 368 participants. In a small subset of participants consenting (n=30), a stool sample is obtained at baseline, 6 months, and 24 months to study the microbiome in an exploratory analysis.

**Study extension:** We propose to assess the domain specific composite for memory (including both RBANS immediate and delayed memory scale) yearly up to three years after the completion of the trial as the primary outcome for the extension study. Secondary outcomes include changes in other cognitive domains that include (1) attention/executive domain (assessed through reaction time/information processing speed/conceptual shifting/selective attention assessed in RBANS, (2) working memory (RBANS).

**3.4 Treatment**

**3.4.1 Placebo vs. 2 grams of DHA:** The intervention will be algal-derived triglyceride DHA-S (DSM, Columbia, Maryland) administered as 5 capsules per day, (total of 2 grams per day). Each capsule DHA-S has ~40-45% DHA and 2-3%EPA, and ~16% DPAn-6, In the general population, around 2 grams of DHA achieves saturable levels in the plasma [[58](#_heading=h.2fk6b3p)]. Placebo capsules were identical in size and appearance and consist of 50% corn oil/50% soy oil obtained from DSM.

**3.4.2 Vitamin B and DHA Brain Transport:** Recent data suggest that adequate vitamin B and folic acid levels are necessary for transport of DHA to the brain, reviewed in section 2.4 [[59](#_heading=h.upglbi), [60](#_heading=h.3ep43zb)]. We propose to provide all participants with a Vitamin B complex supplement (Homocysteine regulator, Solgar). It consists of Vitamin B 6 (50 mg), Folic acid (400 ucg), Vit B12, (500 ucg), TMG (1 g) and P5P (6 mg) taken as two capsules per day. The choice of this supplement is based on the past study linking DHA, Vit B levels, with cognition and brain volumes in the VitaCog and FACIT trials [[59](#_heading=h.upglbi), [60](#_heading=h.3ep43zb)].

**3.4.3 FDA Compliance:** DHA is fish oil that is available over the counter and does not require prior FDA approval. i.e., an IND, if it is not being tested to treat a specific medical illness (which it is not in this study).

**4 RECRUITMENT**

**4.1 Sources of Participants**

This project will recruit a total of 160 participants under the direction of Drs. Hussein Yassine, Lon Schneider, and Howard Hodis.

| ***Table 3: Existing source of participants*** | | | | | |
| --- | --- | --- | --- | --- | --- |
|  | ***Number*** | ***Age*** | ***Sex*** | ***# ɛ4 heterozygotes*** | ***# ɛ4 homozygotes*** |
| *ELITE [*[*61*](#_heading=h.1tuee74)*]* | *643* | *>40* | *Females* | *165* | *23* |
| *WISH [*[*62*](#_heading=h.4du1wux)*]* | *350* | *>44* | *Females* | *80* | *1* |
| *BVAIT[[63](#_heading=h.2szc72q)]* | *506* | *>40* | *Females and males* | *88* | *11* |
| *ADRC AD offspring* | *107* | *>40* | *Males and females* | *47* | *12* |

We obtained a planning pilot award from the Alzheimer’s Association in 2015 to build the infrastructure for recruitment and identify participants eligible for this study. We identified three primary recruitment sources where we can access existing information on age, sex and APOE genotype to greatly enhance efficiency of screening (**Table 3**). The first recruitment source is through the Atherosclerosis Research Unit (ARU) led by Drs. Hodis and Mack. Information on 2252 participants is available from the completed ELITE, WISH, BVAIT and VEAPS trials [[61](#_heading=h.1tuee74), [63-65](#_heading=h.2szc72q)]. The second source is the families of the USC ADRC participants. We identified 107 participants from the UDS/NACC population and the ADRC participant registry who have a diagnosis of MCI or AD, and we obtained IRB approval to mail them our study flyer. Our goal is to reach out to their families to reach possible APOE ɛ4 carriers. In addition to the above resources, we are also listed on the ADRC research participant crib sheet and on the ADRC research website. We have also posted study flyers in primary care clinics and USC affiliated community health centers to recruit from the general population within 30 miles of USC. To augment recruitment, we plan to run one week of radio ads/year. The third source of participants is using Keck EMR query using I2B2. We will mail participants between the ages of 55-80 and with one cardiovascular risk factor, an invitation letter with the study flyer. Thus, we have access to a large number of individuals likely to meet recruitment criteria. Our study design is illustrated in **Figure 3**.

**4.2 Recruitment and Screening**

The Programmer/Analyst identifies patients who meet the inclusion/exclusion criteria from the ARU database. The recruiter/coordinator sends letters to potential participants describing the purpose of the study and providing individuals the opportunity to opt out of being contacted. Eight weeks later, contact information for individuals who do not opt out is entered into the study recruitment database. For recruiting family members of ADRC participants, individuals with MCI and AD will also be entered into the recruitment database, and will be mailed brochures that explain our study. For the ADRC crib sheet, the ADRC research coordinator will identify potential patients who meet inclusion/exclusion criteria from the Network’s databases and clinical records. The ARU recruiter and coordinator will identify participants eligible for this study. The trial coordinator will contact potential participants from all sources to describe the study and determine eligibility and willingness to be screened, and will schedule participants that satisfy our inclusion/exclusion criteria.

During COVID-19 pandemic, all screening visits will take place either online or over the phone. Platforms that will be used during this time include Zoom, FaceTime, and WhatsApp. Screening will take place in a single visit to USC ARU. Individuals who satisfy our inclusion/exclusion criteria will provide informed consent for participation in the study and will complete ω-3 questionnaire. If their DHA consumption/day is <200 mg and MMSE ≥ 25, they will complete a detailed medical history and study questionnaires. When APOE genotype is not known, salivary samples will be obtained. Results will be reviewed by the study coordinator and Dr. Yassine to confirm eligibility.

After screening, eligible participants will start with a run-in period of 2 weeks. The study team will provide individual dietary instructions regarding the ω-3 dietary intake: not to exceed 2 fish or seafood servings per week, and to avoid ω-3 supplemented alimentary products such as some milks, juices, breads, and eggs. Subjects will also be asked to limit their alcohol consumption during the protocol (≤ 2 regular drinks per week are allowed). In addition, subjects will not be allowed to ingest ω-3 supplements or natural health products during the protocol. Individuals will be asked to return for baseline cognitive testing, MRI, and randomization. Participants cannot take additional ω-3 supplements (pills) after randomization during the two year of this study. ω-3 fortified milk or diets are allowed after the two-week screening during the two-year study, and ω-3 intake will be assessed during their 6 months visits. Participants are encouraged not to exceed 200 mg per day of DHA intake based on the DHA intake questionnaire.

**4.3 Informed Consent**

During COVID-19 pandemic, consenting will be obtained using REDCap’s e-Consent feature and USC DocuSign. Consent is obtained in a quiet setting prior to the initiation of any study procedures. The subject is provided adequate time to read and understand the written consent and ask questions as necessary. If they choose, they may take additional time to discuss the study with family and/or physicians outside the clinic setting. Participants are not consented until they demonstrate adequate understanding of all aspects of the study and consent process. A copy of the consent is given to the subject. A model consent form appears in Appendix 2.

In the event a significant protocol change occurs, the informed consent will be adjusted appropriately, and sites will submit the revised documents to their Institutional Review Board (IRB) for approval. Local IRBs will determine whether it is necessary to re-consent participants.

Dr. Yassine will submit to the investigators (Hodis) stamped IRB approval letters and current copies of all consent forms prior to study initiation, and annually thereafter. These records will be maintained by the Dr. Yassine as a local archive.

**4.4 Baseline Testing and Follow-up Visits**

During COVID-19 pandemic, screening and baseline visits will be conducted online or through the phone. Platforms that will be used during this time include Zoom, FaceTime, and WhatsApp. Screening and baseline visits and 6 follow-up visits will be conducted at USC ARU. During the screening visit, vitals, medications, eligibility, and blood work will be obtained. The second visit is planned for the baseline MRI to assess brain volume and connectivity. Participants will be randomized 1:1 into DHA or placebo. To monitor compliance, coordinator (Cordova) will call participants every two weeks in the first two months and then monthly afterwards*.* In addition, participants will be asked to return at 3 months to complete the ω-3 intake questionnaire, and to count the DHA capsules. At the 6 months visit, participants are requested to come complete a ω-3 questionnaire and the battery of cognitive tests. At 12 and 16 months, participants will be requested to come for repeating cognitive testing, the ω-3 questionnaire, and for obtaining vitals (weight, height, and blood pressure). At 24 months, participants will be requested to repeat cognitive testing, the ω-3 questionnaire, for obtaining vitals, and for repeat MRIs and the optional adipose tissue biopsy. During COVID-19 pandemic, participants may receive supplements or study information through the mail. Pill dispensing will occur at randomization, 6-, 12-, and 18-month visits. A fasting stool sample will be obtained at baseline, 6 and 24 months after randomization will be obtained in a subset of participants (optional).

**Study extension:**

After the 24 months, participants will be consented and invited to participate in yearly neuropsychology testing for up to three years to follow up with our trial with DHA about the long-term effects on cognition.

Participants who completed the trial will be called and asked about their interest in participating in the extension study. Participants currently finishing the trial will be asked during their last in person or phone study visit.

**4.5 Randomization**

Following informed consent and determination of eligibility into the LP or no-LP arm, participants are randomized in a 1:1 allocation to two grams of DHA or placebo per day in identically appearing capsules and treated over two years. Within each LP and no-LP arm, randomization is stratified by APOE4 carrier status (E4, no E4) and recruitment site (2 levels), with a blocking factor that is not revealed to investigators. Participant selection to LP and no-LP arms is not randomized and is based on consenting to the LP procedure. The randomization sequence was developed and is monitored by trial statisticians. After verification of trial eligibility and randomization strata, randomization is completed by an unblinded trial statistician. All researchers and participants are blind to the specific intervention.

**4.6 Masking**

The study coordinator will mask the study bottles. Both investigators and participants will be masked to the intervention. Participants will be offered to be told what they were randomized to at the closure of the study at 24 months.

**5 PARTICIPANT MANAGEMENT**

**5.1 Overview**

Study treatment interventions are described above (Section 3.4). They will be provided free of charge to the study participants. Beyond providing DHA and monitoring for any adverse events, the research study will not assume responsibility for the medical care of the participant. Medical problems will be referred to the participant’s primary care provider. For those participants who do not have a primary care provider, the study staff will help find appropriate medical care.

- 1. **DHA Distribution**

DHA will be obtained from the same distributor (as DSM Nutritional Products). The investigator will organize and maintain inventory. *S*ufficient medication is kept on hand to allow medication distribution for the 2-year period. Expiration dates of all medications will be double checked and documented on arrival to the investigator and at the time of distribution to participants to ensure that the provided supplies will not expire during the current treatment interval.

- 1. **Adherence Measures**

Adherence is documented, monitored, and addressed as part of participant care and management and to monitor whether procedures are being implemented and followed. Adherence refers to all study procedures – not only taking the prescribed amount of study drug, but also attending scheduled study visits and completing procedures at those visits. The goal is to maintain high levels of adherence in all participants. Primary adherence measures will address visit attendance and completion of study procedures and medication adherence. Participants will be instructed to bring all study medication (including empty bottles) to study visits for assessment of medication compliance by capsule count. Adherence will be assessed at every visit (See Table 5 below) and in monthly calls in the first three months.

- 1. **Retention**

Retention refers to efforts to prevent participant dropout or withdrawal from the study. It is critically important to successfully engage and retain participation over the course of the trial. For purposes of sample size estimation, investigators have predicted a withdrawal rate of 20% during the 2-year treatment periods and have adjusted sample size to meet study requirements. However, lower rates of attrition are desirable.

The study PI (Yassine) is primarily responsible for monitoring participant attrition and initiating team conferences for retention strategies or dropout recovery rates. Participants missing scheduled visits will be contacted within 24 hours of the scheduled visit to reschedule and discuss strategies to improve compliance. If the subject can’t be contacted, a certified letter will be sent to encourage continuation in the trial even if they no longer wish to take the study medication.

1. **DATA COLLECTION**

**6.1 Pre-screening and Screening Eligibility Measurements**

*Prescreening*: Prescreening for potential eligibility is done at USC ARU. Participants are eligible to continue screening procedures if they satisfy the inclusion/exclusion criteria. Letters can be sent to potential participants describing the purpose of the study and providing individuals the opportunity to opt in to be contacted. Potential participants are contacted directly. The investigators will interview potential participants by phone to determine potential eligibility and interest in the study.

*Screening (table 4)*: Participants who meet pre-screening eligibility criteria are invited to CSC or HMRI to provide informed consent and complete screening, including a directed history and a physical exam, as well as clinical and dietary supervision. All participants would be advised to consume less than two portions fatty fish/wk (e.g., of salmon, herring, or sardines), and fill dietary and exercise questionnaires. Cheek swabs will be obtained to assess APOE genotype status if unknown. If APOE status is known, consent will be obtained to get this information from past studies.

| **Table 4: Data Collection during Screening** | | |
| --- | --- | --- |
| **STEP** | **TEST / PROCEDURE** | **COMMENTS** |
| 1. **Pre-screening** | Query of existing participant records | Contact if age >55, meets inclusion/exclusion criteria |
|  | Phone Screening questionnaire | Initial review of eligibility |
| 1. **Screening** | Neuropsychology questionnaires | Eligibility: inclusion/exclusion criteria |
|  | Targeted history and physical exam and questionnaires (medications, DHA consumption and exercise questionnaire), saliva collection for ApoE genotyping | Eligibility evaluation, consenting and enrollment |
|  | Blood work | For biomarkers |

- 1. **Main Study Procedures**

The timing of major study procedures is illustrated in table 5. Data to determine main study outcomes using the LP will be obtained at baseline and at 6 months, which will be defined as the end of 24 weeks after initiation of DHA. Cognitive testing will be done at baseline, 6 months, 12, 18 and 24 months. MRI will be obtained at baseline and at 24 months. Participants will be offered an optional adipose tissue biopsy to assess DHA stores. After completion of the two-year trial, participants will be invited to participate in a 3-year study extension trial, to study the long-term effects of the study intervention on cognition. The yearly cognitive testing will consist of questions and answers relating to attention, memory, and perception. This cognitive testing can take approximately 1 hour to complete.

**Lumbar puncture:** The participant will be placed in the lateral decubitus position (asked to lie on his or her side with knees curled up toward the chest). The L3-4 or L4-5 interspace will be infiltrated with 1% lidocaine to provide local anesthesia. The LP will be performed atraumatically with a 24g bullet-tip Sprotte spinal needle (when available or equivalent LP kit if not available) and 28cc of cerebrospinal fluid will be withdrawn with sterile syringes. Use of the 24g Sprotte atraumatic spinal needle is recommended by published guidelines on prevention of post-lumbar puncture headache and greatly reduces frequency of post-lumbar puncture headache (<1%). This technique has been used with very low incidence of adverse events. The participant will remain in bed for one hour following the LP to reduce the chance of post-LP headache and not exertion for 48 hours.

**Adipose tissue biopsy:** Adipose tissue is the largest store of omega-3 fatty acids in the body. We are interested in understanding how much DHA is stored in adipose at baseline and after the intervention.  At the baseline and at the 2-year visit, participants will be given an option of undergoing an abdominal subcutaneous fat biopsy following a 12-hour fast. A sample will be collected from an area in the lower quadrant of the abdomen, (10–12 cm from the umbilicus) using a 16-gauge needle without incision. A Yale needle will be attached to a 20 mL syringe filled with sterile saline and will be passed through the subcutaneous fat several times while applying negative pressure. This procedure takes 15 minutes. A local anesthetic is provided to reduce any discomfort. Samples are immediately frozen. The results are not provided to the participants. The adipose tissue biopsy will be done by the study PI (Dr. Yassine).

| **Table 5. Timetable of Study Events for Each Participant** | | | | | | | | | | | | | |
| --- | --- | --- | --- | --- | --- | --- | --- | --- | --- | --- | --- | --- | --- |
| **Visit number** |  | **1** | **2** |  | **3** |  | **4** |  | **5** |  | **6** |  | **7** |
| **Visit and Call Schedule** | **Call 1 Prescreen** | **Screen** | **Baseline** | **Call 2-6** | **Month 3** | **Call 7-8** | **Month 6** | **Call 9-13** | **Month 12** | **Call 14-18** | **Month 18** | **Call 19-23** | **Month 24** |
| **Demographics** | x |  |  |  |  |  |  |  |  |  |  |  |  |
| **Eligibility** | x |  |  |  |  |  |  |  |  |  |  |  |  |
| **Informed Consent** |  | x |  |  |  |  |  |  |  |  |  |  |  |
| **APOE Genotype** |  | x |  |  |  |  |  |  |  |  |  |  |  |
| **Medical History** |  | x |  |  |  |  |  |  |  |  |  |  |  |
| **Vital Signs** |  | x |  |  |  |  | V6 |  | V7 |  | V8 |  | V9 |
| **Medications** |  | x |  |  |  |  | V6 |  | V7 |  | V8 |  | V9 |
| **DHA Food Frequency Questionnaire** |  | x |  |  | V5 |  | V6 |  | V7 |  | V8 |  | V9 |
| **Exercise Questionnaire** |  | x |  |  |  |  | V6 |  | V7 |  | V8 |  | V9 |
| **Clinical Dementia Rating** |  |  | V1 |  |  |  |  |  |  |  |  |  |  |
| **FAQ** |  | x |  |  |  |  |  |  | V7 |  |  |  | V9 |
| **MMSE** |  | x |  |  |  |  |  |  |  |  |  |  | V9 |
| **Weschler Logical Memory II** |  | x |  |  |  |  |  |  |  |  |  |  |  |
| **Randomization** |  |  | V1 |  |  |  |  |  |  |  |  |  |  |
| **Dispense meds** |  |  | V1 |  | V5 |  | V6 |  | V7 |  | V8 |  |  |
| **RBANS** |  |  | V1 |  |  |  | V6 |  | V7 |  | V8 |  | V9 |
| **Color Trails Test (CTT)** |  |  | V1 |  |  |  | V6 |  |  |  |  |  |  |
| **Cogtrack*** |  |  | V1 |  |  |  | V6 |  | V7 |  | V8 |  | V9 |
| **LP** |  |  | V2 |  |  |  | V6 |  |  |  |  |  |  |
| **Adipose Tissue*Biopsy (optional)** |  |  | V4 |  |  |  |  |  |  |  |  |  | V12 |
| **Gut microbiome (optional)** |  |  | V2 |  |  |  | V6 |  |  |  |  |  | V10 |
| **Brain MRI** |  |  | V3 |  |  |  |  |  |  |  |  |  | V11 |
| **Blood Draw** |  | x |  |  |  |  | V6 |  |  |  |  |  | V10 |
| **Stool collection** |  |  | V1 |  |  |  | V6 |  |  |  |  |  | V10 |
| **Urine collection** |  | x |  |  |  |  |  |  |  |  |  |  |  |
| **Biomarker Blood** |  | x |  |  |  |  | V6 |  |  |  |  |  | V10 |
| **Adverse events** |  |  |  | C1 | V5 | C2 | V6 | C3 | V7 | C4 | V8 | C5 | V9 |
| **Compliance: Call** |  |  |  | C1 |  | C2 |  | C3 |  | C4 |  | C5 |  |
| **Compliance: Capsule Count** |  |  |  |  | V5 |  | V6 |  | V7 |  | V8 |  | V9 |

V1-12: visits 1-12.

C1-5: calls 1-5

* Cogtrack and the adipose tissues biopsies were not performed.

- - 1. **Dietary and Physical Activity Advice During Pre-screening:** After recruitment, the study will start with a run-in period of 2 weeks. Individual dietary instructions will be given by Dr. Yassine with specific advice to consume less than two portions fatty fish/wk (e.g., of salmon, herring, or sardines), and to complete dietary and exercise questionnaires. Participants will be asked to follow these dietary recommendations and maintain their body weight stable throughout the protocol. Additional specifications, regarding the PUFA dietary intake, include avoiding enriched ω-3 PUFA alimentary products (form will be given) such as some milks, juices, breads, eggs. In addition, participants will not be allowed to take vitamins or natural health products during the protocol.

**6.2.2 Fasting Blood and Stool Samples (see Table 3):** DHA levels will be measured at fasting blood at baseline and at 6 months. Blood DHA levels will be measured at Huntington Medical Research Institute by mass spectrometry in collaboration with Drs. Michael Harrington and Alfred Fonteh, as previously described [[37](#_heading=h.2iq8gzs)]. Aliquots of fasting blood will be stored for subsequent measurement of biomarkers (glycosylated hemoglobin, lipid profile, homocysteine level, basic metabolic panel). A blood sample for DNA will be collected for APOE genotyping during prescreening if APOE genotype is not known. A urine sample may be obtained during the screening visit to measure urine lipids and biomarkers. A stool sample may be obtained during baseline, 6, and 24 month visits in a subset of participants.

**6.2.3 APOE Genotyping:** If the APOE genotype is not known, DNA of the participants will be extracted from Saliva or from 200ml of whole blood obtained during prescreening (QIAmp DNA Blood Mini Kit; Qiagen). The DNA fragment containing the apoE gene (APOE) sequence will be amplified by PCR using genotype specific primers. After amplification, the DNA fragment will be assessed using qPCR.

**6.2.3 Physical Measures:** Height will be measured with a stadiometer. Weight will be measured with a calibrated electronic scale.

**6.2.4 Questionnaires:** Participants will complete the PUFA intake questionnaire, exercise, and RBANS, sleep (Pittsburg Sleep Quality Index), depression (Geriatric Depression Scale) and Cogtrack Questionnaires.

**6.2.5 Brain Imaging:** Brain imaging will consist of two MRI scans at baseline and 24 months. MRIs will be conducted at USC. Subjects will be consented and escorted to the scanning suite for image acquisition. We will use a standard MRI protocol similar to ADNI-2 for its scans (see <http://adni.loni.usc.edu/methods/mri-acquisition/>). Participants will receive the following: T1-weighted whole brain volumetric (MPRAGE), T2 FLAIR, T2*, DTI, rf-MRI, 3DASL, DCE MRI, and T2 (FSE). Participants will not be excluded from the study if they do not complete the MRIs. Participants who do not complete the imaging but agree to the LP can still be enrolled in the study.

**6.2.6 Safety Measures:** DHA is relatively safe. Adverse events (AE) will be surveyed at each visit using a standardized questionnaire.

**6.2.7 GUID:** A Global Unique Identifier (GUID) will be generated for each participant, in addition to a study ID.  Each GUID unambiguously identifies a research study participant across different research studies within USC without exposing protected health information (PHI).  When investigators pool data together from multiple studies, GUID's provide the means to detect subjects who participate in more than one study.

We use a GUID generator from LONI to convert participant PHI (e.g., birth name, birth date, place of birth) into irreversible hash codes. Only the hash codes will be sent to the GUID authority operated by LONI, which will assign GUID's and store them along with other non-identifiable Prevent E4 participant information in the clinical database. The participant's PHI will not be stored electronically.

**7 SAFETY / HUMAN SUBJECTS PROTECTION**

This study will be conducted in compliance with the protocol and all applicable regulatory requirements. The participating sites have Federal-wide Assurance with the Office for Human Research Protections, and they follow local HIPAA regulations. Prior to study initiation, the protocol and the informed consent documents will be reviewed and approved by the IRB at each participating site and by an independent Data and Safety Monitoring Board (DSMB). Any amendments to the protocol or consent materials must be approved by the DSMB and the IRBs before implementation.

**7.1 Data and Safety Monitoring Board (DSMB)**

The study will be monitored by an independent DSMB organized by the NIA. The DSMB will consist of three members, at least one of whom will have experience with the use of fish oil, lumbar puncture risks, and cognitive changes with aging. No investigator involved in the trial will be a member of the DSMB. The initial task of the DSMB will be to review the protocol, procedures manual, and consent form to identify any necessary modifications. If modifications are necessary, revisions will be reviewed by the DSMB prior to its recommendation on initiation of the project. The DSMB, based on its review of the protocol, will identify the data parameters and format of the information to be regularly reported. The DSMB will be informed of the occurrence of any serious adverse events and immediately notified of fatal or life-threatening events. The DSMB may at any time request additional information from Dr. Yassine. The DSMB will initially be provided with data blinded to treatment status, but they may request unblinded data if there is a safety concern. Based on the review of safety data, the DSMB will make recommendations regarding the conduct of the study. These may include amending safety-monitoring procedures, modifying the protocol or consent, terminating the study, or continuing the study as designed. The discussions and decisions of the DSMB will be summarized in written reports and provided to the NIH program director and Dr. Yassine. The summary DSMB reports will also be distributed to our local IRB, where applicable. The DSMB will meet in person or by conference call twice per year. Using a DSMB, there is substantial oversight and case review to alert the investigators to any safety issues that may arise in a timely manner

- 1. **Safety Review Plan and Monitoring**

1. *Justification of Sample Size*: See Section 9.3.
2. *Stopping Rules*: The investigators may suggest terminating the study at any time for safety reasons. Safety reasons include any serious side effects reported in section 8.
   1. **Confidentiality**
3. *Protection of Participant Privacy*: Participants’ names are linked to study IDs only on a single recruitment database and only certified project personnel are authorized to have access to this database.
4. *Database Protection*: All hard-copy files are stored in locked cabinets within locked offices at the clinical centers. Primary electronic data are entered into and maintained on a secure USC server with two-level password protection against non-project personnel. The server is backed up daily and automatically. At the end of the study, all hard copy records will be kept in a secure locale for a period dictated by local IRB and Institutional policies, as well as FDA regulations, whichever is longest.
5. *Confidentiality during AE Reporting*: AEs and SAEs are recorded on data collection forms and reported to the Coordinating Center without personal identifiers. Identifiers on accompanying documents (such as medical records) are removed before submission to the USC and any subsequent transfer to the IRB.

**7.4 Expected Side Effects**

The study will use DHA capsules taken with meals.

DHA at 2 grams is safe for most people when taken by mouth, and available over the counter. DHA can cause nausea, intestinal gas, bruising, and prolonged bleeding at larger doses (>6 grams). Fish oil containing DHA can cause fishy taste, belching, nosebleeds, and loose stools. Taking DHA with meals can often decrease these side effects.

**7.5 Risk Management**

**7.5.1 DHA supplementation:** To minimize the risk of gastrointestinal side effects, DHA supplements will be taken with meals.

**7.5.2 Lumbar Puncture:** There is the risk of headache, bleeding, and infection following lumbar puncture. The risk will be minimized by doing the procedure while the patient is recumbent, using sterile techniques, and local anesthesia at the site of the injection. The participant will remain in bed for one hour following the LP to reduce the chance of post-LP headache. Drawing blood or cerebrospinal fluid (CSF) may cause pain in the days following the blood or CSF drawing, and the participant may develop a bruise. Minor bleeding may occur at the time of insertion or withdrawal of the needle. Gentle pressure over the puncture site for two to three minutes should prevent or minimize this occurrence. The participant may become dizzy or faint when fluid is drawn. There is also a small risk of local infection at the puncture site or formation of a small blood clot at the site of needle insertion.

When available, will use the 24g Sprotte atraumatic bullet tip spinal needle. If not available by manufacturer, we will use the equivalent similar needle. Use of the Sprotte needle has been recommended in the American Academy of Neurology practice parameter on post-lumbar puncture headache [[66](#_heading=h.meukdy)]. Both the literature and our experience in performing LPs with the Sprotte 24g needle indicate that we may anticipate a low risk of post-LP headache of less than 1%.

**Risk management for LP:** Women of childbearing potential will have a negative urine pregnancy test before undergoing any LP procedures. Pain at the needle insertion site will be minimized by adequate local anesthesia with subcutaneous injection of 1% lidocaine. We will use an atraumatic 24g Sprotte spinal needle which is associated with a reduced risk of adverse events, including post-LP headache. The Sprotte needle has been recommended in published guidelines for reducing risk of post-LP headache.

We reduce the risk of CSF leak by having the participant lie in bed for one hour after the procedure, having them increase their fluid intake, and by instructing participants not to engage in any strenuous activity for the 48 hours following the procedure. All investigators performing LPs are very experienced in performing this procedure in these subject populations.

We will provide a snack of chips and a Mountain Dew (or other caffeine-containing beverage) to participants after the LP. Providing participants something salty and something with caffeine lowers the risk of mild-moderate headaches. Participants will also be provided lunch after the LP.

Lastly, if the participant wishes, the participant’s spouse, other family member, or friend may be present in the room during the LP, and is able to help keep the participant relaxed and comfortable.

Other discomforts of an LP include nerve irritation and allergy to the local anesthesia used. If the participant has a very bad allergic reaction, they can die. Some things that happen during an allergic reaction that could be a sign or symptom of a life-threatening allergic reaction (anaphylaxis) are:

- a rash
- having a hard time breathing
- wheezing
- a sudden drop in blood pressure (making you feel dizzy or lightheaded)
- swelling around the mouth, throat, or eyes
- a fast pulse
- sweating

In case of an emergency, we will call 911.

**7.5.3 Adipose Tissue Biopsy:** To minimize the risk of pain, participants will receive a local anesthetic injection. The area will be cleaned with 100% alcohol to minimize any risk of infection.

- 1. **HIPAA – Protection of Patient Information**

All data collected in the process of pre-screening, screening, and conducting the proposed research will be stored and maintained locally and centrally in compliance with HIPAA regulations. Access to study data will be secure with limited, password-protected access. Only study personnel who are IRB approved will have access to data collected as part of these studies. Data collected locally for the study will be stored on a server that is maintained with the highest stringency of protection.

**8 ADVERSE EVENT REPORTING**

An adverse event is defined as any medical problem experienced by a study participant whether or not considered intervention-related by the clinical center staff. The timely and complete reporting of adverse events is a critical requirement in the conduct of this trial.

**Definition of Serious Adverse Events**

1. The event results in an inpatient hospitalization (any overnight stay associated with an admission).
2. The event results in the prolongation of a hospital stay.
3. The event results in permanent or severe disability.
4. The event results in death.
5. The event results from an overdose (either accidental or experimental) of the study medication.
6. The event is life-threatening.
7. Treatment is required to prevent a serious adverse event, as defined above.
8. Thrombus or embolus.
   1. **Non-serious Adverse Events**

Non-serious adverse events are all AEs which do not meet the above criteria for “serious”. We will monitor the participants for non-serious AEs. Fish oil can rarely lead to diarrhea, urinary tract infections, falls, dizziness, agitation, and hospitalization.

- 1. **Reporting Adverse Events**

AEs will be ascertained in an unbiased manner using standard questions that are identical and administered identically to participants. To accomplish this, AEs will be reported on a standard form that is completed by the study staff at each study visit. Targeted non-serious AEs (e.g., bruises) are ascertained. AEs also include any significantly abnormal physical finding identified on examination (such as bruises) and any significantly abnormal laboratory result obtained on the patient between visits or at the time of the visit.

In order to facilitate timely reporting of SAEs, the clinical center staff who learns of an SAE must enter the Serious Adverse Event Report prior to the close of the following business day and bring it to the attention of the PI. SAEs are reported to the DSMB and NIA within 24 hours of event being reported to the investigator

- 1. **Tracking Adverse Events**
- *Serious Adverse Events:* SAEs will be monitored by the investigators.
- *Non-serious Adverse Events*: Non-serious AEs are tabulated by the primary investigator in the source document.
- *Safety Monitoring:* The investigators will monitor the development of any side effects.

## Emergency Unmasking

Treatments will be unmasked in case of emergencies. The IRB will be informed. Participants will be asked to stop taking any study interventions.

**9 STATISTICAL CONSIDERATIONS**

All analyses will be conducted under the intention-to-treat principle using the treatment as assigned to each subject, and using all available data from all participants. We will assess the distributional characteristics of all variables and will adjust the analysis accordingly by employing transformations or robust (model free) or nonparametric (distribution free) methods.

**9.1 Study Aims**

The primary endpoint is the change in CSF DHA/AA at 6 months in the LP subset, the secondary outcome is the change in brain imaging outcomes after 24 months in all study participants, and the exploratory outcomes include the change in cognitive outcomes and plasma biomarkers after 24 months in all study participants and stool biomarkers after 24 months in a small subset of study participants.

**Study Extension:** We propose to assess the domain specific composite for memory (including both RBANS immediate and delayed memory scale) yearly up to three years after the completion of the trial as the primary outcome for the extension study. Secondary outcomes include changes in other cognitive domains that include (1) attention/executive domain (assessed through reaction time/information processing speed/conceptual shifting/selective attention assessed in RBANS, (2) working memory (RBANS)

**9.2: Mechanistic Biomarkers**

**9.2.1 Association of DHA Uptake with BBB integrity.** BBB integrity will be assessed by the CSF/plasma albumin quotient, CSF fibrinogen and Soluble Platelet-Derived Growth Factor Receptor β (sPDGFRβ). The latter is a marker of pericyte function that is altered in cognitively healthy adults at risk of cognitive decline [[67](#_heading=h.36ei31r)]. We anticipate that APOE4 carriers will demonstrate defective BBB integrity that associates with decreased DHA delivery to CSF. Information obtained will support basic studies into the functionality of lipid transporters (such as MFSD2A) with APOE4 status.

**9.2.2 Association of DHA Uptake with CSF Aβ42 Levels, plasma Aβ42/40 and cPLA2 Activity**: CSF Aβ42, Tau/pTau, and cPLA2 activity will be assessed before and after the intervention. Based on our previous study [[68](#_heading=h.1ljsd9k)], we expect lower CSF Aβ42 levels (defined as <500 pg/mL) will associate with greater CSF cPLA2 activity. We expect these changes to associate with less CSF DHA change and a lower brain volume/connectivity in response to DHA supplementation.

**9.2.3 Changes in CSF and Plasma Eicosanoids, Fatty Acids and Oxylipins after the Intervention:** We will also assess changes in other CSF lipids with a particular focus on EPA (ω-3), AA (ω-6, precursor of inflammatory signaling with increased uptake in AD brain [[69](#_heading=h.45jfvxd)]) to understand whether the defects in fatty acid metabolism associated with APOE4 status are restricted to DHA or extend to other fatty acids. We anticipate that greater CSF cPLA2 activity will be associated with greater CSF AA concentrations.

**9.2.4 Neuroinflammatory CSF Biomarkers:** The molecular biomarker assessment will be performed by the biomarker’s laboratory at the Zilkha Neurogenetic Institute under Dr. Zlokovic (letter enclosed). We will evaluate biomarkers of inflammation, neuronal injury, and injury of other cell types within the neurovascular unit (NVU). Molecular analyses of CSF will use an ultrasensitive battery of ~30 (0.5ml CSF) simultaneously measuring cell-specific biomarkers. These evaluate i) endothelial and pericyte vascular injury and BBB breakdown; ii) vascular repair and angiogenesis; iii) astrocyte injury response; iv) mitochondrial injury; v) immune and inflammatory response; vi) white matter damage; and vii) AD-related neuronal injury.

**9.2.5 Plasma and CSF DHA Phospholipid Distribution and Plasma Homocysteine Levels:** DHA in phospholipid fractions (PC and PE) together with homocysteine levels will be measured in plasma. Following extraction of lipids from sample, the sample will be separate into lipid classes (LPC, PAF, PC, PE/PI, NL) using TLC or HPLC and the collected fractions of interest will undergo acid hydrolysis of GP classes (add deuterated DHA std) followed by GC/MS.

**9.3 Sample Size/Power Calculations**

**Sample size:** The original sample size estimation for the DHA trial was based on the primary outcome of 6-month change in CSF DHA/AA in the LP arm. Sample size was estimated to test an interaction hypothesis between DHA intervention and APOE4 genotype (i.e., that the DHA intervention effect on CSF DHA levels will differ by APOE4 genotype) at 80% power. The interaction effect size (standard deviation of interaction mean effects, divided by pooled between subject SD) of 0.25 relates to a 50% difference in the DHA effect in APOE4 positive compared to APOE4 negative individuals. The required sample size of 32 subjects per DHA/APOE4 cell (128 total) was increased to a sample size of 160 to accommodate an anticipated 20% dropout. Since initial measures of the dropout rate increased to 30% during trial conduct due to the COVID pandemic, the original sample size of 160 would provide reduced power of 74.6% to detect this interaction effect size. To obtain 80% power considering the 30% dropout, the sample size was increased to 184 (46 per cell) for the LP arm of the trial.

The secondary and exploratory trial outcomes will be assessed in the combined sample (LP and no-LP arms). The original sample size estimate of 320 participants (160 in LP arm and 160 in no-LP arm, with 80 APOE4 participants in each arm) was based on a detectable effect size of 0.5 SD for the main effect of DHA compared to placebo on cognitive change among APOE4 participants, with a 20% dropout. In the MIDAS randomized placebo-controlled trial evaluating DHA in persons 60 and older with age-related cognitive decline {Yurko-Mauro, 2010 #3065}, the effect size for 24-week change in verbal memory (delayed recall) was 0.3 SD units. Assuming a roughly double effect size among APOE4 positive participants, will require 45 subjects per group (for an effect size of 0.6 SD units), or 64 subjects per group (for a more conservative effect size of 0.5 SD units). The initial measures of the dropout rate increased to 30% during trial conduct due to the COVID pandemic. To achieve 80% power considering a 30% dropout, we obtained NIH, DSMB and IRB approval to increase the sample to 368 (184 for the LP arm and 184 for the no-LP arm).

**9.4 Data Analysis**

Dr. Mack will lead the data analysis for this project, supervising the data analyst.

Baseline demographics and clinical, laboratory, and questionnaire data will be characterized and compared between randomized groups using frequency tabulations for binary/categorical variables and means (SD)/medians (inter-quartile range) for continuous variables. Baseline characteristics of participants in the LP vs no-LP arms and in those who complete and do not complete the study will also be compared. Initial analyses on trial outcomes will follow the intent-to-treat (ITT) principle by which participants are analyzed according to their randomized group, using complete data (participants who complete trial follow-up outcome measures). The primary outcome of CSF DHA/AA will be evaluated in the LP arm using a general linear model that includes main effects of randomized treatment, the APOE4 carrier and clinical site stratification factors, and the interaction between APOE4 stratum and treatment group. Sensitivity analyses will (1) use multiple imputation procedures on the primary outcome that will involve analyses of all randomized subjects, (2) limit analysis to “compliant” subjects (defined as at least 80% capsule count over the trial period). Secondary and exploratory outcomes, including changes in blood and CSF lipid and inflammation biomarkers, cognition, structural and functional connectivity by MRI and will be analyzed in the entire sample using the same general linear model; additional covariates will include an indicator variable for LP vs. no-LP arm and baseline variables that differ between the two LP arms. Analysis of secondary outcomes will not adjust for multiple comparisons (except for voxel-wise analyses). If there is a significant group difference found (p<0.05) in the primary or secondary outcomes (carriers vs. non-carriers), we will conduct analyses to identify potential mediation of the treatment effect.

**10 DATA PROCESSING AND MANAGEMENT**

- 1. **Data Management System**

We will use REDCap (http://project-redcap.org) as our primary tool for data capture. REDCap is a secure, web-based application that is free through the CTSA consortium. It provides: 1) an intuitive interface for data entry (with data validation); 2) audit trails for tracking data manipulation and export procedures; 3) automated export procedures for data downloads to common statistical packages such as SAS; 4) procedures for importing data from external sources. REDCap has been adopted by many CTSAs across the country. All USC study personnel have access to REDCap through CTSA consortium. A data dictionary and web-based data entry system will be developed which includes data validation algorithms according to case report forms (CRFs). Data from non-CRFs such as laboratory and imaging will be merged by unique study IDs for data analyses.

**10.2** **Data Transfer**

Newly entered and imported clinical center data on REDCap are monitored and managed by Dr. Mack and the trial data manager. Coded data with no patient identifiers containing the CSF and blood DHA levels will be shared with the investigators and stored using REDCap.

**10.3 Quality Control**

Range checks, inter-item checks, cross-table checks, and double data entry verification are used where appropriate to ensure accurate data entry. DHA in capsules from DSM will be measured at HMRI to verify concentrations. Specific quality control procedures are run to check for missing, incorrect, and questionable values immediately after they are entered. Reports with the necessary patient identifying information and the problem values are printed and sent to the clinical research coordinator for correction. When returned, corrected values are entered and checked again for consistency with other items. The goals are to make quality control a continuous process, to make the turnaround time between error detection and correction as short as possible, and to document any changes made to the database.

**10.4 Back-up, Data Security, and Confidentiality**

To assure participant confidentiality, names are linked to study IDs only on a single recruitment database and only limited project personnel are authorized to have access to this database. Only study IDs will be used in all other study databases (e.g., for tracking and merging). All data are maintained on a secure USC server with two-level password protection against non-project personnel. Servers are automatically backed up daily. Backup procedures include twice-weekly system backup, daily incremental backup, and off-site disaster recovery backup. Security procedures include logon and link password protection, and for internet access, separate Web servers which use SSL and encryption algorithms. Virus and malware protection software is used on all computers and is updated on an hourly basis. All portable computers employ full disk encryption. Both USC and HMRI computing facilities provide support in the event of a disaster. Access to the server and databases is secured by use of login user accounts and passwords. Remote access is granted only to authorized users and is accomplished using a secure virtual private network (VPN). Appropriate filtering/firewall setup is used to prevent unauthorized access.

**10.5 Tracking Study Progress**

The purpose of tracking reports is to keep the collaborative group informed of study progress, and to report special problems and resolutions. Reports will be produced regularly by the Data Center, as directed by the DSMB.

Tracking reports include the following types of information:

- screening and enrollment (versus goal), by age, genotype
- tables describing adherence to the study protocol (attendance, intervention compliance, dropout, protocol deviations, completion of trial outcomes)
- database inventory
- progress of analysis and manuscripts

**10.6 Archiving and Study Close-out Including Repository**

At the end of the study, after all data has been received and edited, the database is archived in computer readable format, including readme documentation files, files of study documents (such as forms annotated with variable names, protocols, and manuals of procedures), data files in the form of excel files and input statements, data dictionaries, and program code documenting any derived variables.

**11 STUDY ADMINISTRATION**

**11.1** **Training and Certification**

Prior to being allowed to recruit participants, each investigator will have certification criteria, including satisfactory participation in clinical research training, and Dr. Yassine will supply the IRB approval letter, stamped informed consent forms, and completion of conflict-of-interest policy by all investigators.

Throughout the study, any new staff will be trained at the appropriate clinical center where appropriate. Clinic staff will re-certify annually on identified procedures. Records of certifications and training will be maintained at the clinical sites.

**Conflict of Interest Policy**

The investigators are required to disclose any financial or related interest that could present an actual conflict of interest or be perceived to present a conflict of interest. Disclosure is required to protect each individual’s reputation and career from potentially embarrassing or harmful allegations of inappropriate behavior, and to protect the integrity of Study research. Conflict of interest forms are kept on file at the CoC, and all conflicts are declared at each study group meeting.

**11.2** **Publications and Presentations Policy**

The results of this study will be published. To coordinate dissemination of data from this study, a publication committee will be formed. The committee will consist of the Protocol Committee, interested Principal Investigators, and appropriate USC staff. The committee will solicit input and assistance from other Investigators as appropriate and adhere to all USC Publications Policies.

**11.3 Repository for Storage and Distribution of Data and Samples**

At the end of the study, de-identified research data and samples of blood will be stored at the Yassine lab. The Repository collects, stores, and distributes biological samples and associated data from people with many kinds of disorders, from unaffected family members, and from other healthy people.

**12 STUDY TIMELINE**

The study will be conducted over a period 7 years after all approval is in place.

**13 COMPENSATION**

**Participant Compensation.** Participants will be compensated for their participation. Participants will receive $25 for screening visit, $50 for each MRI completed, $50 for each NP visit completed, and $50 for the optional adipose tissue biopsy and $100 for each LP visit. We will assist with transportation.

The participant will receive 50$ compensation and travel assistance for the yearly visits to study the long-term effects of the study on cognition.

**14 LITERATURE CITED**

1. Lambert, J.-C., et al., *Meta-analysis of 74,046 individuals identifies 11 new susceptibility loci for Alzheimer's disease.* Nature genetics, 2013.

2. Bu, G., *Apolipoprotein E and its receptors in Alzheimer's disease: pathways, pathogenesis and therapy.* Nature Reviews Neuroscience, 2009. **10**(5): p. 333-344.

3. Corder, E., et al., *Protective effect of apolipoprotein E type 2 allele for late onset Alzheimer disease.* Nature genetics, 1994. **7**(2): p. 180-184.

4. Gong, J.-S., et al., *Novel action of apolipoprotein E (ApoE): ApoE isoform specifically inhibits lipid-particle-mediated cholesterol release from neurons.* Molecular neurodegeneration, 2007. **2**(1): p. 9.

5. Vance, J.E. and H. Hayashi, *Formation and function of apolipoprotein E-containing lipoproteins in the nervous system.* Biochim Biophys Acta, 1801. **2010**.

6. Cunnane, S.C., et al., *Fish, docosahexaenoic acid and Alzheimer’s disease.* Progress in Lipid Research, 2009. **48**(5): p. 239-256.

7. Yassine, H.N., et al., *Association of Docosahexaenoic Acid Supplementation With Alzheimer Disease Stage in Apolipoprotein E epsilon4 Carriers: A Review.* JAMA Neurol, 2017.

8. Yassine, H.N., et al., *DHA brain uptake and APOE4 status: a PET study with [1-11 C]-DHA.* Alzheimer's Research & Therapy, 2017. **9**(1): p. 23.

9. Vandal, M., et al., *Reduction in DHA transport to the brain of mice expressing human APOE4 compared to APOE2.* Journal of neurochemistry, 2014. **129**(3): p. 516-526.

10. Nock, T.G., R. Chouinard-Watkins, and M. Plourde, *Carriers of an apolipoprotein E epsilon 4 allele are more vulnerable to a dietary deficiency in omega-3 fatty acids and cognitive decline.* Biochim Biophys Acta, 2017. **1862**(10 Pt A): p. 1068-1078.

11. Dayton, S., et al., *Composition of lipids in human serum and adipose tissue during prolonged feeding of a diet high in unsaturated fat.* J Lipid Res, 1966. **7**(1): p. 103-11.

12. Conway, V., et al., *Apolipoprotein E isoforms disrupt long-chain fatty acid distribution in the plasma, the liver and the adipose tissue of mice.* Prostaglandins Leukot Essent Fatty Acids, 2014. **91**(6): p. 261-7.

13. Kariv-Inbal, Z., et al., *The isoform-specific pathological effects of ApoE4 in vivo are prevented by a fish oil (DHA) diet and are modified by cholesterol.* Journal of Alzheimer's Disease, 2012. **28**(3): p. 667-683.

14. Chouinard-Watkins, R., et al., *Docosahexaenoic acid prevents cognitive deficits in human apolipoprotein E epsilon 4-targeted replacement mice.* Neurobiol Aging, 2017. **57**: p. 28-35.

15. Quinn, J.F., et al., *Docosahexaenoic acid supplementation and cognitive decline in Alzheimer disease: a randomized trial.* JAMA, 2010. **304**(17): p. 1903-11.

16. Stonehouse, W., et al., *DHA supplementation improved both memory and reaction time in healthy young adults: a randomized controlled trial.* Am J Clin Nutr, 2013. **97**(5): p. 1134-43.

17. Andrieu, S., et al., *Effect of long-term omega 3 polyunsaturated fatty acid supplementation with or without multidomain intervention on cognitive function in elderly adults with memory complaints (MAPT): a randomised, placebo-controlled trial.* The Lancet Neurology.

18. van de Rest, O., et al., *Effect of fish oil on cognitive performance in older subjects A randomized, controlled trial.* Neurology, 2008. **71**(6): p. 430-438.

19. Yurko-Mauro, K., et al., *Beneficial effects of docosahexaenoic acid on cognition in age-related cognitive decline.* Alzheimers Dement, 2010. **6**(6): p. 456-64.

20. Huang, T.L., et al., *Benefits of fatty fish on dementia risk are stronger for those without APOE epsilon4.* Neurology, 2005. **65**(9): p. 1409-14.

21. Barberger-Gateau, P., et al., *Dietary patterns and risk of dementia: the Three-City cohort study.* Neurology, 2007. **69**(20): p. 1921-30.

22. Whalley, L.J., et al., *n–3 Fatty acid erythrocyte membrane content, APOE ε4, and cognitive variation: an observational follow-up study in late adulthood.* The American journal of clinical nutrition, 2008. **87**(2): p. 449-454.

23. Daiello, L.A., et al., *Association of fish oil supplement use with preservation of brain volume and cognitive function.* Alzheimer's & Dementia, 2015. **11**(2): p. 226-235.

24. Yassine, H.N., et al., *The effect of APOE genotype on the delivery of DHA to cerebrospinal fluid in Alzheimer's disease.* Alzheimers Res Ther, 2016. **8**(1): p. 25.

25. Yurko-Mauro, K., D.D. Alexander, and M.E. Van Elswyk, *Docosahexaenoic acid and adult memory: A systematic review and meta-analysis.* PLoS One, 2015. **10**(3): p. e0120391.

26. Abdullah, L., et al., *APOE epsilon4 specific imbalance of arachidonic acid and docosahexaenoic acid in serum phospholipids identifies individuals with preclinical Mild Cognitive Impairment/Alzheimer's Disease.* Aging (Albany NY), 2017. **9**(3): p. 964-985.

27. Mapstone, M., et al., *Plasma phospholipids identify antecedent memory impairment in older adults.* Nat Med, 2014. **20**(4): p. 415-8.

28. da Costa, K.A., et al., *Docosahexaenoic acid in plasma phosphatidylcholine may be a potential marker for in vivo phosphatidylethanolamine N-methyltransferase activity in humans.* Am J Clin Nutr, 2011. **93**(5): p. 968-74.

29. Magret, V., et al., *Entry of polyunsaturated fatty acids into the brain: evidence that high-density lipoprotein-induced methylation of phosphatidylethanolamine and phospholipase A2 are involved.* Biochem J, 1996. **316 ( Pt 3)**: p. 805-11.

30. Selley, M.L., *A metabolic link between S-adenosylhomocysteine and polyunsaturated fatty acid metabolism in Alzheimer's disease.* Neurobiol Aging, 2007. **28**(12): p. 1834-9.

31. Ferrari, C., et al., *How can elderly apolipoprotein E epsilon4 carriers remain free from dementia?* Neurobiol Aging, 2013. **34**(1): p. 13-21.

32. Kivipelto, M., et al., *Risk score for the prediction of dementia risk in 20 years among middle aged people: a longitudinal, population-based study.* Lancet Neurol, 2006. **5**(9): p. 735-41.

33. Morris, M.C., et al., *Association of Seafood Consumption, Brain Mercury Level, and APOE epsilon4 Status With Brain Neuropathology in Older Adults.* JAMA, 2016. **315**(5): p. 489-97.

34. Jansen, W.J., et al., *Prevalence of cerebral amyloid pathology in persons without dementia: A meta-analysis.* JAMA, 2015. **313**(19): p. 1924-1938.

35. Yassine, H.N., *Targeting prodromal Alzheimer's disease: too late for prevention?* Lancet Neurol, 2017. **16**(12): p. 946-947.

36. Yassine, H.N. and L.S. Schneider, *Lessons from the Multidomain Alzheimer Preventive Trial.* Lancet Neurol, 2017. **16**(8): p. 585-586.

37. Fonteh, A.N., et al., *Human Cerebrospinal Fluid Fatty Acid Levels Differ between Supernatant Fluid and Brain-Derived Nanoparticle Fractions, and Are Altered in Alzheimer's Disease.* PloS one, 2014. **9**(6): p. e100519.

38. Morris, J.C., et al., *APOE predicts amyloid-beta but not tau Alzheimer pathology in cognitively normal aging.* Ann Neurol, 2010. **67**(1): p. 122-31.

39. Calon, F., *Omega-3 polyunsaturated fatty acids in Alzheimer’s disease: key questions and partial answers.* Curr Alzheimer Res, 2011. **8**.

40. Yassine, H.N., et al., *Association of Serum Docosahexaenoic Acid With Cerebral Amyloidosis.* JAMA Neurol, 2016. **73**(10): p. 1208-1216.

41. Braak, H. and E. Braak, *Frequency of stages of Alzheimer-related lesions in different age categories.* Neurobiology of aging, 1997. **18**(4): p. 351-357.

42. Burggren, A.C., et al., *Reduced cortical thickness in hippocampal subregions among cognitively normal apolipoprotein E e4 carriers.* Neuroimage, 2008. **41**(4): p. 1177-1183.

43. Witte, A.V., et al., *Long-chain omega-3 fatty acids improve brain function and structure in older adults.* Cerebral Cortex, 2014. **24**(11): p. 3059-3068.

44. Sheline, Y.I., et al., *APOE4 allele disrupts resting state fMRI connectivity in the absence of amyloid plaques or decreased CSF Abeta42.* J Neurosci, 2010. **30**(50): p. 17035-40.

45. Goveas, J.S., et al., *Functional network endophenotypes unravel the effects of apolipoprotein E epsilon 4 in middle-aged adults.* PloS one, 2013. **8**(2): p. e55902.

46. Heise, V., et al., *The APOE varepsilon4 allele modulates brain white matter integrity in healthy adults.* Mol Psychiatry, 2011. **16**(9): p. 908-16.

47. Thomas, J., et al., *Omega-3 Fatty Acids in Early Prevention of Inflammatory Neurodegenerative Disease: A Focus on Alzheimer’s Disease.* BioMed Research International, 2015. **2015**.

48. Persson, J., et al., *Altered brain white matter integrity in healthy carriers of the APOE epsilon4 allele: a risk for AD?* Neurology, 2006. **66**(7): p. 1029-33.

49. Smith, C.D., et al., *White matter diffusion alterations in normal women at risk of Alzheimer's disease.* Neurobiology of aging, 2010. **31**(7): p. 1122-1131.

50. Westlye, L.T., et al., *Effects of APOE on brain white matter microstructure in healthy adults.* Neurology, 2012. **79**(19): p. 1961-1969.

51. Greicius, M.D., et al., *Default-mode network activity distinguishes Alzheimer's disease from healthy aging: evidence from functional MRI.* Proceedings of the National Academy of Sciences of the United States of America, 2004. **101**(13): p. 4637-4642.

52. Heise, V., et al., *Apolipoprotein E genotype, gender and age modulate connectivity of the hippocampus in healthy adults.* Neuroimage, 2014. **98**: p. 23-30.

53. Donohue, M.C., et al., *The preclinical Alzheimer cognitive composite: measuring amyloid-related decline.* JAMA Neurol, 2014. **71**(8): p. 961-70.

54. Soininen, H., et al., *24-month intervention with a specific multinutrient in people with prodromal Alzheimer's disease (LipiDiDiet): a randomised, double-blind, controlled trial.* The Lancet Neurology. **16**(12): p. 965-975.

55. Duff, K., et al., *Diagnostic Accuracy of the RBANS in Mild Cognitive Impairment: Limitations on Assessing Milder Impairments.* Archives of Clinical Neuropsychology, 2010. **25**(5): p. 429-441.

56. Caselli, R.J., et al., *Longitudinal modeling of age-related memory decline and the APOE ε4 effect.* New England Journal of Medicine, 2009. **361**(3): p. 255-263.

57. Kulzow, N., et al., *Impact of Omega-3 Fatty Acid Supplementation on Memory Functions in Healthy Older Adults.* J Alzheimers Dis, 2016. **51**(3): p. 713-25.

58. Arterburn, L.M., E.B. Hall, and H. Oken, *Distribution, interconversion, and dose response of n− 3 fatty acids in humans.* The American journal of clinical nutrition, 2006. **83**(6): p. S1467-1476S.

59. Jernerén, F., et al., *Brain atrophy in cognitively impaired elderly: the importance of long-chain ω-3 fatty acids and B vitamin status in a randomized controlled trial.* The American journal of clinical nutrition, 2015: p. ajcn103283.

60. Durga, J., et al., *Effect of 3-year folic acid supplementation on cognitive function in older adults in the FACIT trial: a randomised, double blind, controlled trial.* Lancet, 2007. **369**(9557): p. 208-16.

61. Hodis, H.N., et al., *Methods and baseline cardiovascular data from the Early versus Late Intervention Trial with Estradiol testing the menopausal hormone timing hypothesis.* Menopause, 2015. **22**(4): p. 391-401.

62. Hodis, H.N., et al., *Isoflavone soy protein supplementation and atherosclerosis progression in healthy postmenopausal women: a randomized controlled trial.* Stroke, 2011. **42**(11): p. 3168-75.

63. Hodis, H.N., et al., *High-dose B vitamin supplementation and progression of subclinical atherosclerosis: a randomized controlled trial.* Stroke, 2009. **40**(3): p. 730-6.

64. Hodis, H.N., et al., *Alpha-tocopherol supplementation in healthy individuals reduces low-density lipoprotein oxidation but not atherosclerosis: the Vitamin E Atherosclerosis Prevention Study (VEAPS).* Circulation, 2002. **106**(12): p. 1453-9.

65. Hodis HN, M.W., Shoupe D, et al. , *Testing the menopausal hormone therapy timing hypothesis: the Early Vs Late Intervention Trial with Estradiol.* Circulation. 2014;130:A13283., 2014.

66. Evans, R.W., et al., *Assessment: prevention of post-lumbar puncture headaches: report of the therapeutics and technology assessment subcommittee of the american academy of neurology.* Neurology, 2000. **55**(7): p. 909-14.

67. Montagne, A., et al., *Blood-brain barrier breakdown in the aging human hippocampus.* Neuron, 2015. **85**(2): p. 296-302.

68. Fonteh, A.N., et al., *Alterations in cerebrospinal fluid glycerophospholipids and phospholipase A2 activity in Alzheimer's disease.* J Lipid Res, 2013. **54**(10): p. 2884-97.

69. Esposito, G., et al., *Imaging neuroinflammation in Alzheimer's disease with radiolabeled arachidonic acid and PET.* Journal of Nuclear Medicine, 2008. **49**(9): p. 1414-1421.

70. Yassine, H.N., et al., *The effect of APOE genotype on the delivery of DHA to cerebrospinal fluid in Alzheimer's disease.* Alzheimers Res Ther, 2016. **8**: p. 25.
